# Celastrol suppresses colorectal cancer via covalent targeting peroxiredoxin 1

**DOI:** 10.1038/s41392-022-01231-4

**Published:** 2023-02-03

**Authors:** Heng Xu, Hongfang Zhao, Chunyong Ding, Defang Jiang, Zijie Zhao, Yang Li, Xiaoyu Ding, Jing Gao, Hu Zhou, Cheng Luo, Guoqiang Chen, Ao Zhang, Ying Xu, Hao Zhang

**Affiliations:** 1 School of Pharmaceutical Science and Technology, Hangzhou Institute for Advanced Study, University of Chinese Academy of Sciences, Hangzhou, 310024 China; 2 Chemical Biology Research Center, State Key Laboratory of Drug Research, Shanghai Institute of Materia Medica, Chinese Academy of Sciences, Shanghai, 201203 China; 3 CAS Key Laboratory of Tissue Microenvironment and Tumor, Shanghai Institute of Nutrition and Health, University of Chinese Academy of Sciences, Chinese Academy of Sciences, Shanghai, 200031 China; 4 Department of Pathophysiology, Key Laboratory of Cell Differentiation and Apoptosis of the Chinese Ministry of Education, Shanghai Jiao Tong University School of Medicine, Shanghai, 200025 China; 5 Pharm-X center, College of Pharmaceutical Sciences, Shanghai Jiao Tong University, Shanghai, 200240 China; 6 School of Chinese Materia Medica, Nanjing University of Chinese Medicine, 138 Xianlin Road, Qixia, Nanjing, 210023 Jiangsu China; 7 University of Chinese Academy of Sciences, No.19A Yuquan Road, Beijing, 100049 China; 8 State Key Laboratory of Oncogenes and Related Genes, Shanghai Cancer Institute, Ren-Ji Hospital, Shanghai Jiao Tong University School of Medicine, Shanghai, 200127 China

**Keywords:** Target identification, Target validation

## Abstract

As a terpenoids natural product isolated from the plant *Thunder God Vine*, Celastrol is widely studied for its pharmacological activities, including anti-tumor activities. The clinical application of Celastrol is strictly limited due to its severe side effects, whereas previously revealed targets and mechanism of Celastrol seldom reduce its in vivo toxicity via structural optimization. Target identification has a far-reaching influence on the development of innovative drugs, and omics data has been widely used for unbiased target prediction. However, it is difficult to enrich target of specific phenotype from thousands of genes or proteins, especially for natural products with broad promising activities. Here, we developed a text-mining-based web-server tool to enrich targets from omics data of inquired compounds. Then peroxiredoxin 1 (PRDX1) was identified as the ROS-manipulating target protein of Celastrol in colorectal cancer. Our solved high-resolution crystal structure revealed the unique covalent binding mode of Celastrol with PRDX1. New derivative compound 19-048 with improved potency against PRDX1 and selectivity towards PRDX2~PRDX6 were synthesized based on crystal structure analysis. Both Celastrol and 19-048 effectively suppressed the proliferation of colorectal cancer cells. The anti-tumor efficacy of Celastrol and 19-048 was significantly diminished on xenograft nude mice bearing PRDX1 knock-down colorectal cancer cells. Several downstream genes of p53 signaling pathway were dramatically up-regulated with Celastrol or 19-048 treatment. Our findings reveal that the side effects of Celastrol could be reduced via structural modification, and PRDX1 inhibition is promising for the treatment of colorectal cancer.

## Introduction

**Fig. 1:**
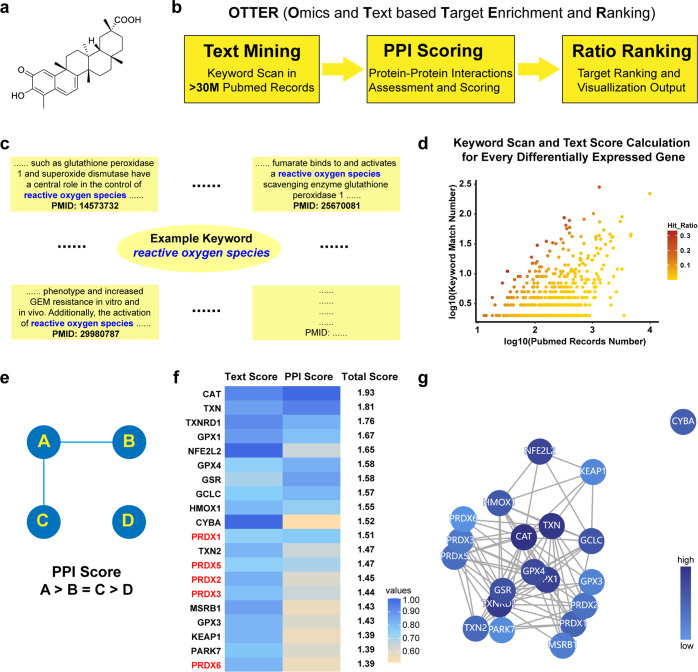
A new computational tool named OTTER for target identification. **a** The chemical structure of Celastrol. **b** The workflow of OTTER consists of three steps, including text mining in PubMed abstracts, scoring with protein-protein interactions, and ranking with visualization. **c** Illustrative diagram for the text mining process of OTTER. Several abstracts containing the keyword “reactive oxygen species” were chosen as an example. For each gene in the list provided by users, the user-defined keyword is scanned in all the PubMed abstracts of this gene. **d** Text mining and scores calculation are performed for every differentially expressed gene in the list provided by users. For each gene, a hit ratio is calculated using the Keyword Match Number divided by the PubMed Record Number. Then a hit ratio is normalized as a Text Score for every gene in the list. **e** The rule used by OTTER for protein-protein interaction assessment. After the calculation of Text Scores, these genes are scored with the protein-protein interaction records from the STRING database. The genes involved in more protein-protein interactions with other genes in the list are given higher PPI Scores. **f** The top-ranking 20 differentially expressed genes according to total scores calculated by OTTER, using the RNA sequencing data from cells treated with Celastrol, and the keyword “reactive oxygen species”. **g** The interactive plot generated by OTTER, using top-ranking 20 differentially expressed genes according to final scores. Colored nodes represent top-ranking genes after enrichment, while lines represent protein-protein interactions between these genes. Nodes with higher scores are colored in darker blue

Celastrol is one of the most promising medicinal natural products isolated from traditional medicines.^1^ As a pentacyclic triterpene (Fig. 1a) isolated from the plant *Thunder God Vine*, Celastrol is widely studied for its pharmacological activities, including anti-tumor, anti-obesity, and anti-inflammatory activities.^2^ Accumulating evidence indicated that Celastrol exhibited effective anti-tumor efficacy against a broad spectrum of cancer cells, including prostate cancer, liver cancer, breast cancer, melanoma, and glioma.^3–7^ Notably, induction of apoptosis and ROS (reactive oxygen species) were frequently observed in various cancer cells treated with Celastrol. For example, Celastrol induced ROS and growth inhibition in bladder cancer cells, while this efficacy was blocked by inhibitors of ROS.^8^ Celastrol induced ROS accumulation, G2/M arrest, and apoptosis in ovarian cancer and glioma cells, while ROS scavenger NAC (N-acetyl-L-cysteine) blocked Celastrol-induced apoptosis.^9,10^ Similar phenotypes were observed in non-small cell lung cancer H1299 and hepatocellular carcinoma Hep-G2 cells.^6^ Celastrol showed effective cytotoxicity on drug-resistant colon cancer cells via ROS-dependent mechanism.^11^

Colorectal cancer is the third most commonly diagnosed malignant cancer and the second leading cause of cancer death globally.^12^ Accumulating evidence indicated that the levels of ROS are commonly higher in various cancer cells, including colorectal cancer cells, than normal cells.^13,14^ ROS play multiple roles in normal cellular physiological processes, while ROS accumulation shows dual roles to cells.^13^ Under physiological conditions, moderate ROS elevation promotes cell proliferation and differentiation, whereas excessive ROS causes oxidative damage to DNA, protein, and lipids. Cells control ROS levels by balancing its generation and elimination with ROS-scavenging proteins such as peroxiredoxins, superoxide dismutases, and glutathione peroxidases.^14^ Peroxiredoxins scavenge ROS via their peroxidase activity, reducing hydrogen peroxide and a wide range of organic hydroperoxides.^15^ Under pathological conditions, ROS elevation is associated with abnormal cancer cell growth and reflects disrupted redox homeostasis. Cancer cells with increased oxidative stress are more vulnerable to damage by further ROS elevation induced by exogenous agents, which can induce apoptosis and cell cycle arrest in cancer cells partly mediated by the p53 signaling pathway.^14,16^ Then, it is feasible to selectively kill cancer cells via ROS manipulating, without causing significant toxicity to normal cells.^14^ The underlying target protein of Celastrol, which is able to manipulate the redox homeostasis in various cancer cells, is promising for the anti-tumor drug discovery and development. However, the relationship between ROS, Celastrol and p53 signaling pathway in colorectal cancer remains undiscovered.

Despite its promising anti-tumor activities, clinical application of Celastrol is strictly limited due to severe side effects.^2^ Previously revealed targets and mechanism indicated that Celastrol exerted anti-tumor efficacy through distinct targets in different cancer cells, while these findings seldom provide comprehensive evaluation at molecular level.^17–19^ Moreover, without crystal structure of Celastrol in complex with its target protein, it is extremely difficult to reduce the toxicity of Celastrol via structural modification. Therefore, to determine whether the side effects of Celastrol could be reduced, as well as to provide rational basis for the structural modification of Celastrol, it is worthwhile to discover and validate the ROS- manipulating anti-tumor target protein of Celastrol.

Target identification and validation is a time-consuming and challenging procedure in drug discovery, especially for natural products with complex or unique chemical structures. Experimental approaches based on binding detection inevitably identify target proteins with irrelevant phenotypes. And it is common for existing computational tools to exploit chemical structures of compounds for target discovery. However, this strategy underestimates the target proteins with few or none inhibitors reported. To facilitate the target discovery of small-molecular compounds for specific phenotype, without chemical structure comparison, we have developed a new knowledge-based computational tool for target enrichment, via text mining combined with protein-protein interactions assessment. Using this tool with the omics data from cells treated with Celastrol, we seek to discover and validate the undiscovered target protein responsible for the ROS-manipulating anti-tumor efficacy of Celastrol.

## Results

### Development of a new computational tool named OTTER for target enrichment

It is a common strategy for existing computational target discovery tools, to compare the chemical structure of active compounds with previously reported inhibitors for known target proteins. However, this strategy is not applicable for the identification of target proteins with few or none inhibitors reported. To facilitate the target discovery not applicable to chemical structure comparison, we have developed a new computational tool named **OTTER** (**O**mics and **T**ext based **T**arget **E**nrichment and **R**anking). Starting from a list of differentially expressed genes identified by omics data, this tool is able to enrich the most relevant genes or proteins for specific pharmacological feature, via exhaustive text mining in all the PubMed abstracts available. In addition to the list of differentially expressed genes, users need to provide a “keyword”, which represents the pharmacological feature observed in cells treated with active compounds. The “keyword” can be any word or phrase that might be mentioned in the abstracts of target proteins. For example, the “keyword” can be *apoptosis, reactive oxygen species, glioma, breast*, etc.

The workflow of OTTER consists of three steps (Fig. 1b). First step, for each differentially expressed gene, OTTER scans the keyword, for example “reactive oxygen species”, in all the PubMed abstracts of this gene (Fig. 1c). Then a text score is calculated for this gene, and this procedure of text mining is performed for every differentially expressed gene in the list provided by users (Fig. 1d). Second step, after text mining and ratio quantification, OTTER also takes into account the protein-protein interactions (PPI) among these top-ranking differentially expressed genes. The more protein-protein interactions observed for one gene, the higher PPI score calculated for this gene (Fig. 1e). Third step, final scores are calculated using the sum of text scores and PPI scores. Then a table of final scores for top-ranking 50 genes are generated, along with interactive plots for visual representation. To assess the performance of this new tool, the RNA sequencing data of several approved drugs with known target protein were collected and tested. For example, the target proteins TOP2A and TOP1 of Doxorubicin^20,21^ ranked 23rd and 12th with the keyword “anticancer” (Supplementary Fig. 1a–c), while the target protein ESR1 of Tamoxifen^22^ ranked 1st with the keyword “breast” (Supplementary Fig. 1d–f).

### Celastrol was identified as a covalent inhibitor of PRDX1

**Fig. 2:**
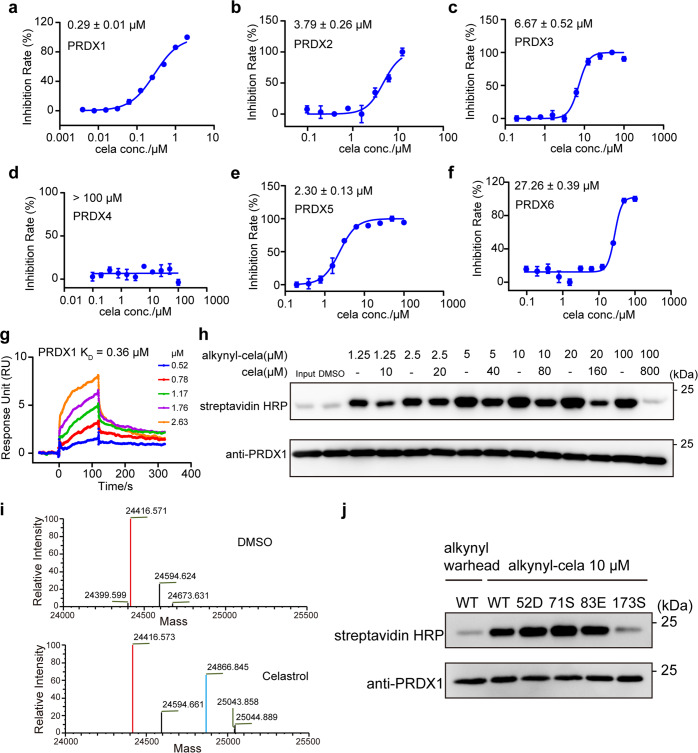
Celastrol was identified as a covalent inhibitor against the peroxidase activity of PRDX1. **a** Peroxidase activity inhibition of PRDX1 with Celastrol incubation. Celastrol was incubated with recombinant PRDX1 for 1.5 h at different concentrations. The inhibition rate was calculated from initial reaction slope of each assay well as shown in Supplementary Fig. 2a. The IC_50_ value of Celastrol against PRDX1 is 0.29 ± 0.009 µM. Peroxidase activity inhibition of PRDX2 (**b**), PRDX3 (**c**), PRDX4 (**d**), PRDX5 (**e**), and PRDX6 (**f**) with Celastrol incubation. Celastrol was incubated with recombinant PRDX2-6 as in (**a**). The inhibition rates were calculated from initial reaction slope in Supplementary Fig. 2b–f for PRDX2~PRDX6. The IC_50_ values of Celastrol are 3.79 ± 0.26 µM for PRDX2, 6.67 ± 0.52 µM for PRDX3, above 100 µM for PRDX4, 2.30 ± 0.13 µM for PRDX5, and 27.26 ± 0.39 µM for PRDX6, respectively. The inhibitions of peroxidase activity by Celastrol were calculated from triplicate experiments. All data is shown in mean ± SEM. **g** The binding affinity of Celastrol with recombinant PRDX1 determined by the SPR assay. **h** Click chemistry labeling of recombinant PRDX1 through alkynylated Celastrol. Covalently bounded PRDX1 was labeled with click chemistry reaction through alkynyl group. “Input” represents PRDX1 without any incubation. “DMSO” represents PRDX1 incubated with DMSO. “Input” and “DMSO” were set as negative control. Click chemistry labeling was blotted with streptavidin HRP, and the amount of PRDX1 in each sample was blotted with anti-PRDX1 primary antibody. **i** Mass spectra analysis of recombinant PRDX1 before and after Celastrol binding. The upper spectra was the sample of PRDX1 incubated with DMSO, while the lower spectra was the sample of PRDX1 incubated with Celastrol for 1.5 h. Main mass peak of ligand-free PRDX1 was shown in red, while peak of PRDX1-Celastrol complex was shown in cyan. The exact mass of Celastrol is 450.277. **j** Click chemistry labeling of recombinant wild type (WT) PRDX1 and four mutants. Alkynylated Celastrol (10 µM) was incubated with WT PRDX1 and four mutants for 1 h and blotted. WT PRDX1 incubated with alkynyl warhead was set as negative control

To provide the gene list required as input by OTTER, RNA sequencing data from HEK-293T cells treated with Celastrol was analyzed, and 6,682 differentially expressed genes were identified. Considering that the elevation of reactive oxygen species was frequently observed in cancer cells treated with Celastrol, the keyword “reactive oxygen species” was chosen to enrich the redox-related target proteins of Celastrol using OTTER. Then it was noted that five peroxiredoxin (PRDX) proteins, including PRDX1, PRDX2, PRDX3, PRDX5, PRDX6, were enriched in the top-ranking 20 genes (Fig. 1f, g). Moreover, several other proteins, including TXN, TXNRD1, and TXN2, are known to cooperate with PRDX proteins.^23–25^ And KEAP1 is known to regulate the transcription of Nrf2 and expression of PRDX proteins.^26,27^ Considering that these clues focus on the human PRDX protein family, the effect of Celastrol on the peroxidase activity of PRDX1~PRDX6 was evaluated with recombinant proteins (Supplementary Fig. 2). The half maximal inhibitory concentration (IC_50_) of Celastrol against the peroxidase activity of peroxiredoxin proteins are 0.29 ± 0.01 µM for PRDX1, 3.79 ± 0.26 µM for PRDX2, 6.67 ± 0.52 µM for PRDX3, 2.30 ± 0.13 µM for PRDX5, and 27.26 ± 0.39 µM for PRDX6, respectively. And no obvious inhibitory effect was observed for Celastrol against the peroxidase activity of PRDX4 (Fig. 2a–f). PRDX1 was preliminarily identified as a potent target of Celastrol from peroxidase activity assay. The binding affinity of Celastrol with PRDX1 protein measured by the surface plasmon resonance (SPR) method is 0.36 μM (Fig. 2g). Previous evidence suggested that Celastrol could form a covalent bond with target protein,^28^ our click labeling assay indicated a synthesized alkynylated Celastrol covalently bound to PRDX1, and this covalent bond could be competed by Celastrol (Fig. 2h).

The difference between mass spectra of PRDX1 with and without Celastrol incubation indicates that one Celastrol molecule covalently bind to one PRDX1 monomer (Fig. 2i). Previous evidence also suggested that Celastrol could form a covalent bond with specific cysteine of target protein.^28^ Therefore, four cysteine single-point PRDX1 mutants were constructed and subjected to click labeling assay with alkynylated Celastrol (Fig. 2j). The results indicate that Celastrol specifically bind to the Cys-173 residue of PRDX1 without affecting the other three cysteines. Taken together, Celastrol was identified as a covalent inhibitor of PRDX1 with potent binding affinity, via addition reaction to the Cys-173 residue of PRDX1.

### Crystal structure of PRDX1 in complex with Celastrol

**Fig. 3:**
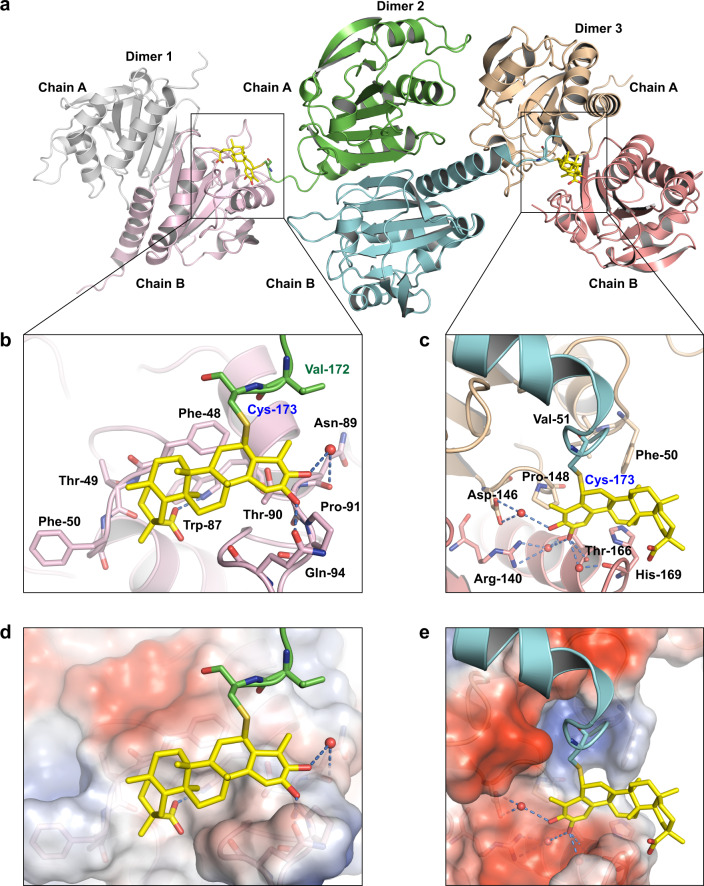
Crystal structure of PRDX1 in complex with Celastrol. **a** Two Celastrol molecules are covalently linked to the two Cys-173 residues of one PRDX1^C52SC83S,1-175aa^ homodimer. Non-covalent binding was observed between these two Celastrol molecules and adjacent PRDX1^C52SC83S,1-175aa^ homodimers. **b***,* **c** The non-covalent binding sites for two Celastrol molecules with adjacent PRDX1^C52SC83S,1-175aa^ homodimers. Celastrol is shown as yellow sticks. Hydrogen bonds are represented as blue dashed lines, and crystal water molecules were represented as red spheres. **d**, **e** Surface representation of the non-covalent binding sites of two Celastrol molecules. Electronegative region is colored in blue, while electropositive region is colored in red. Hydrogen bonds are represented as blue dashed lines, and crystal water molecules are represented as red spheres

Previous study predicted that Celastrol covalently bound to target protein in a stereospecific manner, which might impact the target protein selectivity of Celastrol.^28^ To reveal the stereospecific conjugation and binding mode of Celastrol, the crystal structure of PRDX1 in complex with Celastrol was solved to the resolution of 1.76 Å (Supplementary Table 1). To obtain homogeneous oligomeric and less flexible proteins for crystallization, the Cys-52 and Cys-83 residues were mutated to serine, and the C-tail of PRDX1 (176-199 aa) was truncated. Then it was found that two PRDX1^C52SC83S,1-175aa^ monomers formed a head-to-tail homodimer, and two Celastrol molecules were covalently linked to the Cys-173 residues of the homodimer (Fig. 3a). The *F*o-*F*c electron density map contoured to 2.5σ for Celastrol is intact and clear (Supplementary Fig. 3a). Moreover, the 2*F*o-*F*c electron density map contoured to 1.0σ for Celastrol linked to the Cys-173 residues confirms that Celastrol is a covalent inhibitor of PRDX1 (Supplementary Fig. 3b, c). Since the Cys-173 residue is required for the catalytic activity of PRDX1,^29,30^ the covalent binding of Celastrol with Cys-173 residues inactivates the peroxidase activity of PRDX1.

For each PRDX1^C52SC83S,1-175aa^ homodimer, the covalently linked two Celastrol molecules stretched into and formed non-covalent interactions with adjacent homodimers. Interestingly, the binding modes of two Celastrol molecules with adjacent PRDX1 homodimers are different. Despite Celastrol is a pentacyclic triterpene compound with only four polar atoms, multiple hydrogen bonds were observed between the hydroxyl and carboxyl groups of Celastrol and the surrounding residues. For one Celastrol molecule, direct and water-bridged hydrogen bonds were observed with the Trp-87, Asn-89, Gln-94 residues of adjacent PRDX1 homodimer (Fig. 3b). While for another Celastrol molecule, direct and water-bridged hydrogen bonds were observed with the Arg-140, Thr-166, His-169 residues of another adjacent PRDX1 homodimer (Fig. 3c). In addition to these polar interactions, hydrophobic contacts were also observed between Celastrol and surrounding residues. For one Celastrol molecule, hydrophobic contacts were formed with the Phe-48 and Val-172 residues (Supplementary Fig. 3d), while for another Celastrol molecule, less hydrophobic contacts were observed (Supplementary Fig. 3e).

Based on the superposition of this crystal structure of Celastrol in complex with PRDX1, to the crystal structures of PRDX2-PRDX6 monomers, conformational difference were observed for the binding region of two Celastrol molecules with adjacent PRDX1 homodimers (Supplementary Fig. 3f, g). For example, a hydrogen bond was observed between Celastrol and the Gln-94 residue. While the glutamine residue at this position is not conservative in the human PRDX protein family. Then the relative selectivity of Celastrol against the peroxidase activities of PRDX2~PRDX6 might be partially attributed to the sequence and conformational difference in these binding regions (Supplementary Fig. 3h).

### New derivative compounds of Celastrol were synthesized and evaluated

**Fig. 4:**
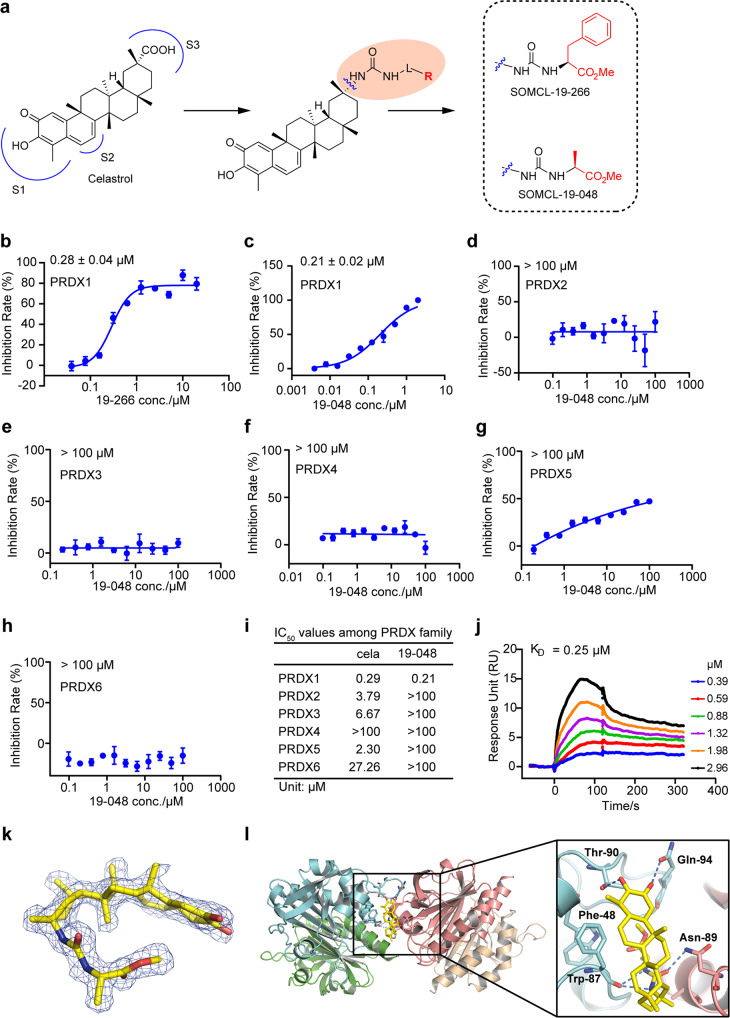
New derivative compound of Celastrol named 19-048 showed improved potency and selectivity. **a** The scheme of synthesis for new derivative compounds of Celastrol. Carboxyl group of Celastrol was substituted by guanidine group. Compound 19-266 and 19-048 were synthesized by guanidine terminal substitution. **b** Peroxidase activity inhibition of PRDX1 by compound 19-266. Recombinant PRDX1 was incubated with different concentrations of compound for 1.5 h. The inhibition rate was calculated from initial reaction slope of each assay well as shown in Supplementary Fig. 4a. The IC_50_ value of compound 19-266 against PRDX1 is 0.28 ± 0.040 µM. All data is shown in mean ± SEM. Peroxidase activity inhibition of PRDX1 (**c**), PRDX2 (**d**), PRDX3 (**e**), PRDX4 (**f**), PRDX5 (**g**), PRDX6 (**h**) incubated with compound 19-048. The inhibition rates were calculated from initial reaction slope of figure S4b-S4g for PRDX1~PRDX6. The IC_50_ value of compound 19-048 against PRDX1 is 0.21 ± 0.02 µM, while the IC_50_ values of compound 19-048 against PRDX2~6 are above 100 µM. The inhibitions of peroxidase activity assay were calculated from triplicate experiments. All data is shown in mean ± SEM. **i** Summary of IC_50_ values for Celastrol and its derivative compound 19-048 against the six proteins from human PRDX family. **j** The binding affinity of compound 19-048 with recombinant PRDX1 determined by the SPR assay. **k** The *F*_O_–*F*_C_ electron density map of compound 19-048 contoured at 2.5 σ. **l** Binding mode of compound 19-048 with PRDX1^C52SC83S,1-175aa^ homodimers. Compound 19-048 is shown as yellow sticks

Since Celastrol showed non-ignorable inhibitions against the peroxidase activities of more than one PRDX proteins, it is not a specific PRDX1 inhibitor. To obtain new PRDX1 inhibitors with improved potency and selectivity, new derivative compounds of Celastrol were synthesized, among which compound 19-266 and 19-048 were synthesized via guanidine substitutions (Fig. 4a). Compound 19-266 inhibits the peroxidase activity of PRDX1 with an IC_50_ value of 0.28 ± 0.04 µM (Fig. 4b, Supplementary Fig. 4a), while for compound 19-048, the IC_50_ value is 0.21 ± 0.02 µM (Fig. 4c, Supplementary Fig. 4b). Moreover, compound 19-048 showed no obvious inhibition against the peroxidase activity of PRDX2~PRDX6 (Fig. 4d–h, Supplementary Fig. 4c–g). Thus, compound 19-048 selectively inhibits the peroxidase activity of PRDX1 among the human PRDX protein family with approximately 500-fold selectivity as compared to Celastrol (Fig. 4i). Consistent with the IC_50_ value, the binding affinity *K*_D_ value measured by the SPR method for compound 19-048 with PRDX1 protein is 0.25 µM (Fig. 4j).

Covalent reactivity of Celastrol and compound 19-048 were characterized with the determination of *K*_inact_ and *K*_i_. For Celastrol the *K*_inact_ value is 0.20 ± 0.019 min^−1^ and the K_i_ value is 0.95 ± 0.086 µM, while for compound 19-048 the *K*_inact_ value is 0.13 ± 0.094 min^−1^ and the *K*_i_ value is 1.88 ± 0.050 µM (Supplementary Fig. 4h, i). Same as Celastrol, compound 19-048 was identified as a covalent inhibitor using the mass spectrometry (Supplementary Fig. 4j). Click labeling assay confirms that compound 19-048 is a covalent inhibitor of PRDX1 (Supplementary Fig. 4k). The click labeling assay using mutant PRDX1 proteins found that the covalent bond was formed between compound 19-048 and the Cys-173 residue of PRDX1, which is consistent with Celastrol (Supplementary Fig. 4l). Thus, the mechanism of covalent reaction for Celastrol with PRDX1 was preserved after the structural modification from Celastrol into compound 19-048.

To compare the binding mode of compound 19-048 with Celastrol, the crystal structure of PRDX1^C52SC83S,1-175aa^ in complex with compound 19-048 was solved to the resolution of 1.81 Å (Supplementary Table 1). Different from Celastrol, only one compound 19-048 was found in each PRDX1 homodimer. Covalent bond was not clearly observed between compound 19-048 and the Cys-173 residue. This might be due to the difference of reactivity between Celastrol and compound 19-048. Then the electron density for compound 19-048 linked with the Cys-173 residue is not as strong as Celastrol. The *F*o-*F*c electron density map contoured to 2.5σ and 2*F*o-*F*c electron density map contoured to 1.0σ for compound 19-048 are intact and clear (Fig. 4k, Supplementary Fig. 4m). Due to the introduction of new chemical groups, compound 19-048 formed more hydrogen bonds with surrounding residues than Celastrol, including Phe-48, Thr-90, Gln-94, and the Asn-89 residue from another PRDX1 homodimer (Fig. 4l, Supplementary Fig. 4n). In terms of this non-covalent binding site shared by Celastrol and compound 19-048, the overall binding modes of two compounds are similar (Supplementary Fig. 4o). However, orientational discrepancy was observed for compound 19-048 as compared to Celastrol, which might contribute to its improved selectivity. Taken together, as compared to Celastrol, compound 19-048 showed remarkable selectivity against the peroxidase activity of PRDX2~PRDX6, as a selective PRDX1 inhibitor.

### Celastrol and compound 19-048 induced cell cycle arrest and apoptosis by increasing ROS in colorectal cancer cells

Increasing evidence suggests that PRDX1 is closely involved in the development of human colorectal cancer by scavenging ROS.^31^ To assess the efficacy of Celastrol and compound 19-048 in colorectal cancer cells, the proliferation of colorectal cancer cells after Celastrol and 19-048 treatment was evaluated. Our results showed that Celastrol and compound 19-048 suppressed the proliferation of SW620 cells with IC_50_ values of 689.57 nM and 528.23 nM respectively (Supplementary Fig. 5a), and suppressed the proliferation of HCT116 cells with IC_50_ values of 893.83 nM and 815.93 nM respectively (Supplementary Fig. 5b). In addition, cell count and viability detected by trypan blue staining further confirmed that Celastrol and compound 19-048 decreased cell number and maintained approximate 80% cells viability at low concentration (0.5 μM), while significantly decreased cell viability at high concentrations (1, 2 μM) in both SW620 and HCT116 cells (Supplementary Fig. 5c–f). We further treated normal human colon mucosal epithelial cell line NCM460 with Celastrol or compound 19-048. The results showed that Celastrol and 19-048 suppressed the proliferation of NCM460 with IC_50_ values of 2661 nM and 2230 nM, respectively (Supplementary Fig. 6a), which were 2–3 folds higher than that of colorectal cancer cells SW620 and HCT116.

PRDX1 is a crucial antioxidant protein to regulate oxidative stress.^32^ To determine whether Celastrol and compound 19-048 impact the cellular redox state via targeting PRDX1, ROS levels were measured by dichlorodihydrofluorescein diacetate (DCFH-DA) probe staining in SW620 and HCT116 cells. After treatment of Celastrol and compound 19-048 at different concentrations, sharp elevations in cellular ROS content were observed as compared to the DMSO control. The ROS-positive rates in colorectal cancer cell SW620 were 61.07% and 74.77%, and in HCT116 were 88.4% and 91.7% after treatment with Celastrol or compound 19-048 for 24 h at 2 μM. Treatment with the antioxidant agent N-acetyl-L-cysteine (NAC)^33^ in SW620 and HCT116 cells completely suppressed the elevated ROS induced by treatment of Celastrol and compound 19-048 (Supplementary Fig. 5g, h). Subsequently, we detected the effects of Celastrol and 19-048 on ROS levels in NCM460 cells. NCM460 cells were treated with 0.5–2 μM Celastrol or compound 19-048 for 24 h, and the intracellular ROS positive rates were 26.5% and 29.13% at 2 μM, respectively (Supplementary Fig. 6b), suggesting that Celastrol and compound 19-048 have less effect on the ROS induction in normal cells than in cancer cells.

Elevated excessive ROS has been reported to increase cell cycle arrest and apoptosis.^34,35^ To explore whether Celastrol and compound 19-048 suppressed the proliferation of colorectal cancer cells via cell cycle regulation, cell cycle distribution based on flow cytometry after compound treatment was analyzed. SW620 cells were arrested in the G2/M phase and apoptosis was induced in a dose-dependent manner, in the presence of Celastrol and compound 19-048 (Supplementary Fig. 5i, j). The same effect was also observed in HCT116 cells (Supplementary Fig. 5k, l). Notably, NAC treatment also completely abolished the cell cycle arrest and apoptosis in SW620 and HCT116 cells induced by Celastrol and compound 19-048 (Supplementary Fig. 5i–l), indicating that the cell cycle arrest and apoptosis induced by Celastrol and compound 19-048 in colorectal cancer cells are ROS-dependent. The DNA damage marker γ-H2AX^36^ was further detected and increased accumulation of γ-H2AX in the nucleus was observed after treatment of Celastrol and compound 19-048 (Supplementary Fig. 7). Together, these results demonstrated that compound 19-048 exhibited similar effects as Celastrol that suppressed the proliferation of colorectal cancer cells in a ROS-dependent manner.

### Celastrol and compound 19-048 suppressed the proliferation of colorectal cancer cells via targeting PRDX1

**Fig. 5:**
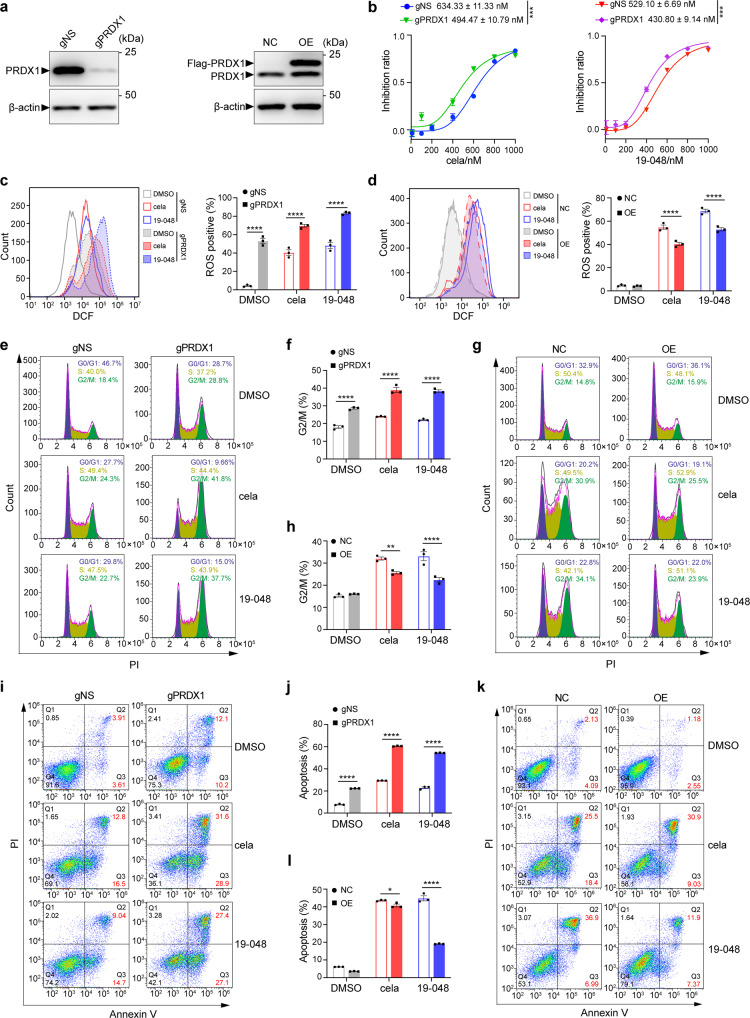
Celastrol and compound 19-048 suppressed the proliferation of colorectal cancer cells via targeting PRDX1. **a** PRDX1 protein level with gPRDX1 (left) or PRDX1 overexpression (right) lentivirus plasmid infection in SW620 cells was analyzed by western blot. Protein names are marked with triangle in the left of corresponding band. Endogenous PRDX1 is marked as “PRDX1”. PRDX1 overexpression fused with an N-terminal flag tag is marked as “Flag-PRDX1”. β-actin was used as internal control. Molecular weights are marked in the right of protein bands. **b** The IC_50_ curves of Celastrol or compound 19-048 against the proliferation of scrambled (gNS) and PRDX1 knockdown (gPRDX1) SW620 cells. Compounds were incubated with cells for 24 h before measurement. ***p < 0.001 by Student’s *t* test. **c** Intracellular ROS level was analyzed by flow cytometry with DCFH-DA staining in gNS and gPRDX1 SW620 cells with 0.5 μM Celastrol or compound 19-048 for 24 h. Quantification of ROS level in each group and statistical analysis was shown in the right histogram. **d** Intracellular ROS level was analyzed by flow cytometry with DCFH-DA staining in non-coding control plasmid (NC) and PRDX1 overexpression plasmid (OE) SW620 cells treated with 1 μM Celastrol or compound 19-048 for 24 h. Quantification of ROS level in each group and statistical analysis are shown in the right histogram. **e**, **f** Cell cycle distribution was detected by flow cytometry with PI staining in gNS and gPRDX1 SW620 cells treated with 0.5 μM Celastrol or compound 19-048 for 24 h. Quantification of cells in the G2/M phase and statistical analysis are shown as (**f**). **g**, **h** Cell cycle distribution was detected by flow cytometry with PI staining in NC and OE SW620 cells treated with 1 μM Celastrol or compound 19-048 for 24 h. Quantification of cells in the G2/M phase and statistical analysis are shown as (**h**). **i**, **j** Apoptotic cells were measured by flow cytometry with Annexin V and PI staining in gNS and gPRDX1 SW620 cells treated with 0.5 μM Celastrol or compound 19-048 for 24 h. Quantification of apoptotic cells (Annexin V^+^) and statistical analysis are shown as (**j**). **k**, **l** Apoptotic cells were measured by flow cytometry with Annexin V and PI staining in NC and OE SW620 cells treated with 1 μM Celastrol or compound 19-048 for 24 h. Quantification of apoptotic cells (Annexin V^+^) and statistical analysis is shown as (**l**). Data are presented as mean ± SEM (*n* = 3). Statistical significance was determined by two-way ANOVA. *p < 0.05, **p < 0.01, ***p < 0.001, ****p < 0.0001

To validate that PRDX1 is the dominant target protein for the cell cycle arrest and apoptosis induced by Celastrol and compound 19-048 in colorectal cancer cells, we generated PRDX1-knockdown (gPRDX1) SW620 cells by CRISPR-Cas9 system, with the scrambled negative sgRNA as control (gNS) (Fig. 5a). Both Celastrol and compound 19-048 showed decreased IC_50_ values in gPRDX1 SW620 cells, suggesting that knockdown of PRDX1 could enhance the sensitivity of colorectal cancer cells to Celastrol and compound 19-048 (Fig. 5b). As compared to the SW620-gNS cells, the accumulation of cellular ROS increased in SW620-gPRDX1 cells, and compound treatment (Celastrol or 19-048) further enhanced the extent of ROS elevation (Fig. 5c). In contrast, overexpression (OE) of PRDX1 in SW620 cells (Fig. 5a) markedly reversed the ROS elevation induced by Celastrol and compound 19-048 (Fig. 5d). Consistent with the regulation of intracellular ROS, increased G2/M cell cycle arrest and enhanced apoptosis were observed after knockdown of PRDX1, while the overexpression of PRDX1 markedly attenuated these effects induced by Celastrol and compound 19-048 (Fig. 5e–l). These results indicate that PRDX1 is the dominant target protein of Celastrol and compound 19-048 in colorectal cancer cells, and the inhibition of PRDX1 leads to elevated intracellular ROS as well as increased cell cycle arrest and apoptosis.

### Inhibition of PRDX1 by Celastrol or compound 19-048 upregulates p53-dependent transcription in colorectal cancer cells

**Fig. 6:**
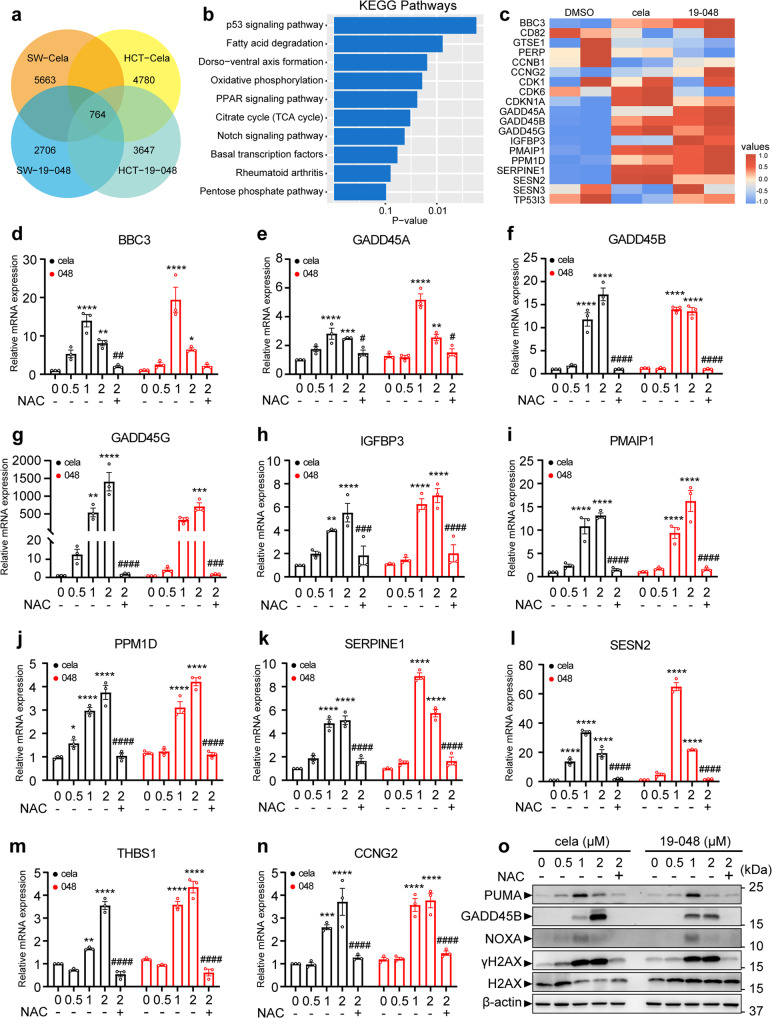
Transcriptome-wide RNA sequencing to identify related signaling pathways and genes affected by Celastrol and compound 19-048 in colorectal cancer cells. **a** Venn diagram for the number of differentially expressed genes from WT SW620 or WT HCT116 cells treated with Celastrol or compound 19-048. Cela, Celastrol; SW, SW620; HCT, HCT116. **b** Statistical histogram for the KEGG pathway analysis using the 764 differentially expressed genes shared by four groups in (**a**). **c** Heatmap of differentially expressed genes from the p53 signaling pathway for SW620 cells after treatment with Celastrol or compound 19-048. **d**–**n** Transcriptional regulation of p53 target genes with Celastrol or compound 19-048 treatment were measured in SW620. Cells were treated with the indicated concentrations (μM) of Celastrol or compound 19-048 for 24 h. Genes were analyzed by qRT-PCR. NAC was used at the concentration of 5 mM. **o** Protein expression level of p53 target genes (GADD45A, NOXA, PUMA) and DNA damage marker (γH2AX, H2AX) were analyzed in SW620. Cells were treated with the indicated concentrations (μM) of Celastrol or 19-048 for 24 h. Protein levels were analyzed by western blot. NAC was used at the concentration of 5 mM. Data are shown from triplicated experiments as mean ± SEM. Statistical significance was determined by two-way ANOVA. *p < 0.05, **p < 0.01, ***p < 0.001, ****p < 0.0001 versus vehicle. ^#^*p* < 0.05, ^##^*p* < 0.01, ^###^*p* < 0.001, ^####^*p* < 0.0001 versus 2 μM Celastrol or 2 μM compound 19-048

To characterize the effect of Celastrol and compound 19-048 in colorectal cancer cells at transcriptome level, the RNA sequencing data from SW620 and HCT116 cells was collected and analyzed. Then 5663 and 2706 differentially expressed genes (DEGs) were identified from SW620 cells treated with Celastrol and compound 19-048, respectively. While 4780 and 3647 DEGs were identified from HCT116 cells treated with Celastrol and compound 19-048, respectively (Fig. 6a). The number of DEGs shared by four treatment groups is 764. Then the Kyoto Encyclopedia of Genes and Genomes (KEGG) pathway enrichment analysis using these shared genes revealed that the p53 signaling pathway was the top-ranking cancer-related signaling pathway (Fig. 6b). The heatmap for dozens of DEGs from the p53 signaling pathway suggested that several target genes of p53 transcription were sharply up-regulated in the presence of Celastrol or compound 19-048 (Fig. 6c).

The p53 signaling pathway is vital in the response to ROS elevation and DNA damage, which further regulates cell cycle arrest and apoptosis.^37,38^ Based on the RNA sequencing data, we identified 21 and 14 DEGs from the p53 signaling pathway in SW620 and HCT116 cells, respectively (Supplementary Fig. 8a). There were 26 DEGs in total, and 9 DEGs were shared in these two groups. RNA expression levels of these DEGs from the p53 signaling pathway were verified after the treatment of Celastrol and compound 19-048. Then 11 genes were up-regulated in SW620 cells after the treatment of Celastrol or compound 19-048, whereas NAC blocked the transcriptional up-regulation of these genes (Fig. 6d–n). Consistent results were also observed in HCT116 cells (Supplementary Fig. 8c–m). The up-regulation of these DEGs were also verified by western blotting (Fig. 6o, Supplementary Fig. 8b). It is known that GADD45B and GADD45G are involved in the regulation of G2/M phase cell cycle arrest,^39–41^ while BBC3 (also named PUMA) and PMAIP1 (also named NOXA) are involved in the regulation of apoptosis.^42^ Recent evidence indicated that PRDX1 could enhance NOXA ubiquitination and degradation, thus inhibiting the apoptosis of colorectal cells.^43^ Meanwhile, the expression levels of p53 target genes were detected in NCM460 cells. The results showed that the expression of NOXA, GADD45B and PUMA slightly changed at 2 μM compounds treatment (Supplementary Fig. 6c). These results suggested that activated p53 downstream genes were involved in the cell cycle arrest and apoptosis induced by PRDX1 inhibition in the presence of Celastrol and compound 19-048.

### Celastrol and compound 19-048 suppressed xenograft tumor growth of colorectal cancer cells in vivo via targeting PRDX1

**Fig. 7:**
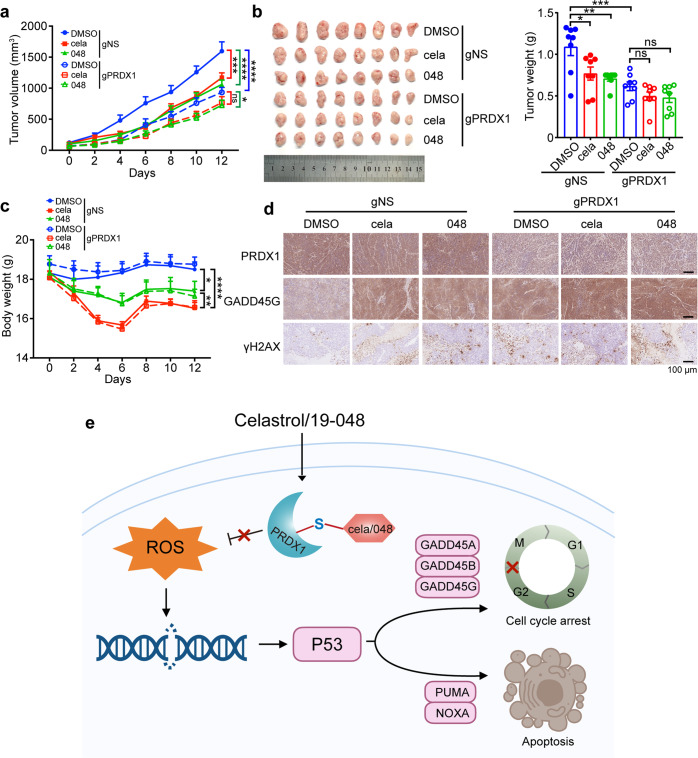
Celastrol and compound 19-048 suppressed xenograft tumor growth of colorectal cancer cells in vivo via targeting PRDX1. Mice bearing SW620 xenograft tumors were treated with Celastrol or compound 19-048 (2 mg/kg, five times per week for two weeks) or vehicle control (*n* = 8 mice per group). **a** Tumor volumes were measured every two days after compound treatment. Right square brackets of statistical significance: red, DMSO vs cela; green, DMSO vs 048, blue, DMSO-gNS vs DMSO-gPRDX1. Statistical significance was determined by two-way ANOVA. **b** Tumors were harvested and photographed (left), statistics analysis of tumors weight were shown (right). Statistical significance was determined by one-way ANOVA. **c** Mice body weights were measured every two days. Statistical significance was determined in gNS group by two-way ANOVA. * represents DMSO vs 048, ** represents cela vs 19-048, *** represents DMSO vs cela. **d** Immunohistochemistry analysis of the xenograft tumors from each group. The protein level of PRDX1, GADD45G, and γH2AX were analyzed. **e** Schematic representation for the mechanism of Celastrol and compound 19-048 targeting PRDX1 to suppress the proliferation of colorectal cancer cells. Data are shown as mean ± SEM. *p < 0.05, **p < 0.01, ***p < 0.001, ****p < 0.0001

To determine whether PRDX1 is the primary target protein of Celastrol and compound 19-048 in vivo, nude mice bearing established SW620-gNS or SW620-gPRDX1 xenografts was used to assess the effect of PRDX1 knockdown on the anti-tumor efficacy of these compounds. When tumors grew to ~50 mm^3^, mice were treated with Celastrol and compound 19-048 at the dose of 2 mg/kg daily for 5 days per week, and the mice treated with DMSO (vehicle) were used as control. The results showed that Celastrol and compound 19-048 suppressed the overall tumor progression of SW620-gNS xenografts with decreasing tumor size, and PRDX1 knockdown group showed similar effect. Further, knockdown of PRDX1 attenuated the inhibition effects of Celastrol or compound 19-048 treatment on tumor growth (Fig. 7a, b). In gNS group, mice body weight significantly decreased with Celastrol treatment as compared to compound 19-048 treatment, and gPRDX1 group showed the same trend (Fig. 7c). Celastrol caused 11.2% body weight loss, whereas compound 19-048 caused 7.3% body weight loss as compared to DMSO control (data calculated from average body weight of DMSO control or compound 19-048 treatment group from gNS and gPRDX1 at day 12). To determine the in vivo toxicity of compound 19-048 and Celastrol, mice acute toxicity test was performed. The percent survival within two weeks was recorded in Supplementary Table 2 and Supplementary Fig. 9a. All the mice died with the treatment of 20 mg/kg Celastrol, while 100% male and 0% female could live with the treatment of 20 mg/kg 19-048. For the lower dose treatment at 10 mg/kg, 25% male and 0% female could live after treatment with Celastrol and 100% mice could live after treatment with compound 19-048. Female mice showed poor survival rate than male mice, possibly due to that female mice had significantly better absorption of Celastrol than male.^44^ Further, we performed chronic toxicity experiment at 2 mg/kg compound treatment for two weeks. The routine blood test demonstrated that the count and proportion of monocyte and neutrophil were higher than the reference intervals in the Celastrol treatment group. As a comparison, these values maintained essentially normal in the compound 19-048 treatment group, showing no significant difference with the DMSO treatment group (Supplementary Fig. 9b, c). These results demonstrated that the in vivo safety of compound 19-048 was significantly improved as compared to Celastrol. Immunohistochemical staining showed that the protein levels of GADD45G and γ-H2AX increased with the treatment of Celastrol and compound 19-048 in SW620-gNS xenografts. As compared to the SW620-gNS xenografts group, reduced protein level of PRDX1 was confirmed in the SW620-gPRDX1 xenografts group. Knockdown of PRDX1 attenuated the up-regulation of GADD45G and γ-H2AX with Celastrol or compound 19-048 treatment, which is consistent with the efficacy assessment for tumor volume and weight (Fig. 7d). These results demonstrate that Celastrol and compound 19-048 suppress the xenograft tumor growth of colorectal cancer cells in vivo, via targeting PRDX1.

## Discussion

Celastrol is considered as one of the most promising medicinal natural products. Previous evidence indicated that Celastrol exerted anti-tumor efficacy through distinct targets in different cancer cells due to tumor heterogeneity. Using our new computational tool OTTER combined with experimental assays, PRDX1 was identified as the primary target protein of Celastrol in colorectal cancer, for its ROS-dependent anti-tumor activity. This finding is basically consistent with previous evidence, while inconsistency exists probably due to the disparity in cancer types or experimental conditions.^17,45^ Considering the accumulating evidence that PRDX1 is involved in various cancer types, and ROS elevation is frequently observed in cancer cells treated with Celastrol, it is likely that PRDX1 might be the primary target protein of Celastrol in other cancer types with ROS abnormality, in addition to the colorectal cancer proved here.

Covalent inhibitors bind with target proteins via covalent reaction with specific residue of target proteins, as well as non-covalent interactions with surrounding residues. Typically, the covalent warhead binding residue and non-covalent binding residues are from the same monomer of target protein. However, the binding mode of Celastrol with PRDX1 homodimers is unique. The covalent binding residue (Cys-173) and non-covalent binding residues are from different PRDX1 homodimers. It is known that PRDX1 homodimers form into decamers in its catalytic cycle,^46^ then this unique binding mode of Celastrol with PRDX1 homodimers is likely to disrupt the decamer formation of PRDX1 homodimers. Therefore, it suggested that the unique binding mode of Celastrol could inactivate the enzymatic activity of PRDX1 potently.

Notably, the covalent addition reaction of Celastrol with target protein was predicted to be in a stereospecific manner.^28^ The crystal structure of PRDX1 in complex with Celastrol solved here confirmed this prediction. In addition, the crystal structure also provided explanation for the selectivity of Celastrol and compound 19-048 against the peroxidase activity of PRDX family proteins. Within the PRDX family, PRDX2~PRDX6 also have exposed catalytic cysteine residues. However, Celastrol showed weak inhibitions against the peroxidase activity of PRDX2~PRDX6, while compound 19-048 showed negligible inhibitions against the peroxidase activity of PRDX2~PRDX6. Then we conclude that the non-covalent binding of Celastrol or compound 19-048 with adjacent PRDX1 homodimers contributs to their stereospecific covalent reaction with the catalytic cysteine residues of PRDX proteins. As a result, the non-covalent binding of Celastrol or compound 19-048 impacts their selectivity within the PRDX protein family.

Our previous report proved that the natural product Adenanthin targeted PRDX1 and PRDX2 to induce differentiation of leukemic cells.^47^ However, it is extremely difficult to extract Adenanthin from plants or via *de novo* synthesis. Moreover, Adenanthin is not a selective PRDX1 inhibitor. These limitations of Adenanthin impeded the exploration of PRDX1 inhibition in various cancer types. Considering the remarkable selectivity in vitro, as well as the improved safety in vivo, compound 19-048 is more suitable than Celastrol and Adenanthin, to serve as a tool compound for the mechanistic study of PRDX1 inhibition. As the in vivo toxicity of Celastrol limits its clinical potential for the treatment of cancer, this new derivative compound 19-048 proved that the toxicity of Celastrol could be reduced via target identification and structural modification.

Recent evidence indicated that PRDX1 could promote the degradation of NOXA, and targeting PRDX1 might be an effective strategy to overcome the resistance of colorectal cancer to DNA damage-inducing chemotherapeutics.^43^ While our results suggest that PRDX1 inhibition alone is sufficient to suppress the proliferation of colorectal cancer. We discovered that inhibiting PRDX1 by Celastrol or its analogs caused ROS elevation and then suppressed the proliferation of colorectal cancer cells, while NAC treatment reversed the apoptotic phenotypes. ROS elevation induced by Celastrol or its analogs treatment subsequently up-regulated apoptosis and cell cycle arrest-related genes of p53 signaling pathway, which were reversed by NAC treatment as well. Therefore, PRDX1 inhibition could up-regulate p53 downstream genes and induce apoptosis with cell cycle arrest in colorectal cancer cells via ROS manipulating (Fig. 7e). These results suggest that PRDX1 inhibition is a promising strategy for the treatment of cancer types that are susceptible to ROS manipulating.

Either transcriptomic or proteomic data is appropriate to provide the list of genes or proteins for OTTER, while this tool has two requirements for users to achieve the best performance. First, the target protein for inquired compound must be in the list of differentially expressed genes or proteins. Then OTTER is not applicable for the cases that the alterations of expression and abundance of target protein for inquired compound are not significant. Second, the selection of “keyword” by users has decisive influence upon the target list ranked by OTTER. Therefore, users are suggested to compare and comprehensively consider the ranked list according to several alternative keywords. On the premise of meeting these requirements, OTTER is able to efficiently enrich the possible target proteins with higher phenotype correlation. Due to its versatility and simple requirement of input, this new computational tool OTTER is expected to be widely used, and to accelerate the target discovery of important natural products or chemical compounds of interest.

## Materials and methods

### Development and web server of OTTER

The web server tool OTTER was developed to facilitate the target discovery research using omics data. This tool inputs user-uploaded list of differentially expressed genes or proteins, and a keyword. Then OTTER scans and ranks the frequency of this keyword in the PubMed abstracts, for each differentially expressed gene or protein in the user-provided list. In addition to the frequency calculation, OTTER also takes into account the protein-protein interactions among all the differentially expressed genes or proteins. Finally, OTTER ranks all the user-provided differentially expressed genes, based on the keyword designated by the users. OTTER is free of charge to academic users, and detailed manuals and examples can be found on its web server (http://otter-simm.com/otter.html).

### Chemicals

Analytically pure powders of Celastrol (Catalog No.CSN16303) were purchased from CSNPharm. And Doxorubicin (Catalog No.HY-15142) and Tamoxifen (Catalog No.HY-13757A) were purchased from MedChemExp. All reagents were obtained commercially and without further purification, Nuclear magnetic resonance (NMR) was used to elucidate the chemical structures based on ^1^H, ^13^C and DEPT-135 NMR experiments. The ^1^H NMR and ^13^C NMR was measured on Varian Plus-400 and Bruker-500 respectively. Mass spectra was measured on Finnigan LTQ. High-resolution mass spectrum was measured on 1290-6545 UHPLC-QTOF.

### RNA-Sequencing

Total RNA was isolated and purified using DNaseI (Takara) and Dynabeads Oligo (dT)25 (Life Technologies) after 24 h. Then purified RNA of 100 ng was used for cDNA library construction, using the NEBNext UltraTM RNA Library Prep Kit for Illumina (NEB). Raw paired-end reads were processed using the Tophat2 software package, with the GRCh38/hg18 Ensembl transcript set. The Cufflinks software package was used to assemble transcripts, estimate their abundance, and identify the differentially expressed genes.^48^ Then the p-values of involved KEGG (Kyoto Encyclopedia of Genes and Genomes) pathways were calculated, using the GeneAnswers package of the Bioconductor project. The raw sequencing data and processed expression files have been deposited to the Gene Expression Omnibus (GEO) under accession GSE198630.

### Plasmid construction and protein expression

The cDNA of wild-type PRDX1,2,3,5,6 and N terminal signal peptide truncated PRDX4 (38aa-271aa) and mutant (C52S, C83S, 1-175aa) PRDX1 were constructed into *Escherichia coli* expression vector pET28a(+). The proteins were expressed in BL21(DE3) strain with LB medium containing 50 µg/ml kanamycin. The protein expression was induced by Isopropyl-beta-D-thiogalactopyranoside (IPTG) at 18 °C. Nickel columns (HisTrap HP, 5 ml, GE Healthcare) were used to purify these his tag recombinant proteins in buffer A (20 mM Tris-HCl, 250 mM NaCl, 1‰ β-mercaptoethanol) and buffer B (20 mM Tris-HCl, 250 mM NaCl, 1 M imidazole, 1‰ β-mercaptoethanol) using AKTA pure system (GE Healthcare). The purified proteins were then subjected to SDS-PAGE and Coomassie blue staining for purity identification. The proteins were then concentrated to 10 mg/ml in buffer C (20 mM Hepes, 100 NaCl).

For the purification of mutant PRDX1 protein used in crystallization, TEV (Tobacco etch virus) protease was used to remove his tag. The TEV protease-treated samples were loaded into nickel column again to separate his tag from mutant PRDX1 protein. The purified mutant PRDX1 were then concentrated to 8 mg/ml for crystallization.

### Trx-TrxR-NADPH coupling peroxiredoxin activity assay

The assay was conducted in 96-well plates as previously described.^47^ Celastrol and its analogs were incubated with PRDX1-6 for 1.5 h at room temperature in each assay well. Then, the mixture solution containing 0.3 µM yeast Trx, 0.15 µM yeast TrxR and 200 µM NADPH was added to assay wells. The reaction was initiated with 100 µM hydrogen peroxide (H_2_O_2_) and detected with Thermo Multiskan FC at 340 nm. For PRDX1-6, the work concentration is 0.6 µM, 1.5 µM, 1 µM, 3 µM, 0.5 µM, 10 µM respectively. The initial reaction’s slope of assay wells containing mixture solution and H_2_O_2_ without PRDXs and compounds (*S*_100%_) were considered as 100 % inhibition of PRDXs’ peroxidase activity. The initial reaction’s slope of assay wells containing mixture solution, H_2_O_2_ and PRDXs without compounds (*S*_0%_) were considered as 0% inhibition of PRDXs’ peroxidase activity. The inhibition rate at different doses of Celastrol and its analogs (*S*_compound_) was calculated from the slope of initial reaction. The inhibition rate (%) was calculated from the following equations: 100 – (*S*_compound_ − *S*_100%_)/(*S*_0%_ − *S*_100%_) × 100. The inhibition rate of Celastrol and its analogs was calculated from three independent experiments. Data were analyzed using GraphPad (version 8.0).

### Click labeling assay

The click labeling method used in this paper refers to these researches.^49,50^ Alkynylated compounds were incubated with recombinant proteins for 1 h in buffer C (20 mM Hepes, 100 NaCl). For some PRDX1 wild type samples, unalkynylated compounds were pre-incubated with proteins for 0.5 h before incubating with alkynylated compounds. The work concentration of proteins is 1 µM. After compounds incubation, the protein samples were subjected to click reaction procedures through terminal alkynyl group as previously described.^49,50^ Protein samples were resolved with SDS-PAGE and blotted with streptavidin HRP (1:10000, Thermo Scientific) and anti-PRDX1 antibody (8499, 1:1000, Cell Signaling Technology).

### Mass spectra assay

Protein samples (3 mg/ml) were incubated with equivalent molar ratio compounds in buffer C (20 mM Hepes, 100 NaCl) for 1.5 h before mass spectra procedures. For mass analysis, protein samples were diluted to 1 mg/ml with 0.1% formic acid. 2 mg of each protein sample was used for LC/MS run. Regular sets of LC/MS instruments and analysis of protein mass were employed as previously described.^51^

### Surface plasma resonance (SPR) assay

The experiment was conducted with Biacore T200 instrument. Recombinant wild type PRDX1 protein was coupled on CM5 chip (Cytiva). Celastrol and 19-048 were firstly dissolved in DMSO as 20 mM stock, then diluted in PBS buffer as mobile phase flowing through PRDX1 coupled chip. Dissociation constant was calculated in kinetic analysis mode using Biacore evaluation software. The final figure was displayed using GraphPad (version 8.0).

### Crystallization and structure determination

The crystal of mutant PRDX1 protein (PRDX1^C52SC83S,1-175aa^) was grown in well solution buffer containing 8% Tacsimate pH 8.0 (v/v) and 20% PEG3350 (v/v) for 7 days at 18 °C. 1 µl protein solution was mixed and incubated with 1 µl well solution in sitting drop plates. Briefly, Celastrol or 19-048 were dissolved in well solution at 100 µM respectively. The well solutions containing 100 µM compound were soaked with ligand free crystals of PRDX1 for at least 15 days at 18 °C. Before X-Ray data collection, the crystals were transferred into cryo-protectant solution (containing 8% Tacsimate pH 8.0 (v/v), 20% PEG3350 (v/v), 22% glycerol) and flash frozen into liquid nitrogen. X-ray diffraction data was collected at the BL19U1 beamline of Shanghai Synchrotron Radiation Facility (SSRF), integrated using the XDS package,^52^ and scaled using the Scala module of the CCP4 software.^53^ The structure was determined by molecular replacement using the Phaser module^54^ of the Phenix software,^55^ with the dimer of PRDX1 (PDB code: 4XCS) as the search template. Then the initial model after molecular replacement was fixed using the ARP/wARP module^56^ of CCP4 and Coot.^57^ The atomic coordinates of Celastrol was determined according to the electron density omit map, using the LigandFit module^58^ of Phenix. The final model was adjusted in Coot and finished after cycles of refinement in Phenix. The statistics for crystal data collection and refinement are summarized in Table S1. The atomic coordinates and structure factors files have been deposited to the Protein Data Bank (PDB) with accession number 7WET and 7WEU for Celastrol and compound 19-048, respectively.

### Cell lines and culture

SW620, HCT116 cell lines were purchased from Cell Bank of Chinese Academy of Sciences, Shanghai. Both cell lines were cultured in RPMI-1640 medium supplemented with 10% FBS and 1% penicillin-streptomycin in a humidified incubator at 37 °C and 5% CO_2_.

### CRISPR-Cas9

To generate PRDX1 knockdown cell lines, gRNA targeting CACCGTCAATACACCTAAGAAACA was cloned into the vector pHBLV-U6-gRNA-EF1-CAS9-PURO from HanBio. The gRNA, psPAX2, and pMD2·G plasmids were mixed and co-transfected into HEK293T cells using Lipofectamine 2000 (Thermo Fisher Scientific). Medium with lentiviruses was collected and filtered through 0.45 μm filters at 48 h after transfection. Lentiviruses was used to infect the indicated cells with 8 μg/mL polybrene (Sigma-Aldrich) for 2 days. The infected cells were selected in medium containing 2 μg/mL puromycin (Sigma-Aldrich) for 3 days, and PRDX1 protein expression level were examined by western blot.

### CCK-8 assay

SW620 or HCT116 cells were seeded into 96-well plate overnight at 5000 cells per well or 4000 cells per well respectively. The culture medium was replaced with medium containing varying concentrations of Celastrol or 19-048 for 24 h. Cell numbers were assessed using the CCK-8 reagent at OD 450. The IC_50_ values were calculated by fitting the data points with the dose-response function in GraphPad.

### Measurement of ROS

The cells were seeded in 6-well plates overnight and treated with Celastrol or 19-048 for 24 h, with or without 5 mM NAC. The cells were harvested and washed with serum-free medium, followed by incubation with DCFH-DA for 20 min at 37 °C in the dark. DCFH-DA was deacetylated by intracellular esterase to a non-fluorescent compound, which was later oxidized by intracellular ROS to the fluorescent DCF. DCF fluorescence was detected using flow cytometer and data was analyzed using FlowJo software (version 10.4).

### Cell cycle assay

After treated with Celastrol, 19-048 or DMSO (Vehicle) for 24 h, the cells were harvested and fixed with 75% cold ethanol overnight at −20 °C. The cells were incubated with RNase I (10 μg/mL) in PBS at 37 °C for 30 min and then stained with PI (50 μg/mL) for 15 min at room temperature. Samples were measured by flow cytometry and data was analyzed by FlowJo software.

### Cell viablity and apoptosis assays

After treated with Celastrol, 19-048 or DMSO (Vehicle) for 24 h, both adherent and floating cells were harvested. Total cell number and viability were counted with trypan blue staining. To measure apoptosis, cells were harvested and followed by flow cytometric analysis using the Annexin V-FITC / PI apoptosis detection kit according to the manufacturer’s protocol. Cell apoptosis was analyzed by FlowJo software.

### Immunofluorescence analysis

Cells were seeded on coverslips and treated with Celastrol or 19-048 for 24 h. After washed with PBS, cells were fixed with 4% paraformaldehyde for 15 min and permeabilized with 0.3% Triton X-100 for 15 min. Cells were blocked with 5% BSA for 1 h at room temperature, followed by incubating with γH2AX antibody overnight at 4 °C and AlexaFluor 488-conjugated donkey anti-Rabbit antibody for 1 h at room temperature. DAPI was used to mark cell nucleus. The cells were visualized by fluorescence microscopy.

### Real-time quantitative PCR

For quantitative analysis of gene expression, total RNA was extracted from the cell samples using Trizol reagent (Invitrogen) according to the manufacturer’s instructions. RNA was treated with DNase I (Promega) and complementary DNA was synthesized using random primers (Takara) and M-MLV Reverse Transcriptase (Promega, Fitchburg, WI). The cDNA was amplified in SYBR Green PCR Master Mix (Applied Biosystems, Foster City, CA). The primers used in quantitative real-time PCR are listed in Supplementary Table 3. β-actin was used as an internal control.

### Western blot analysis

Cells treated with Celastrol or 19-048 were lysed in 1× SDS loading buffer. Cell lysates were fractionated by SDS–PAGE and transferred to nitrocellulose membrane (Bio-Rad, Richmond, CA). After blocked in 5% nonfat milk, the membrane was incubated overnight at 4 °C with the indicated specific primary antibodies, followed by horseradish peroxidase (HRP)-linked secondary antibody (Cell Signaling Technology, Beverly, MA) for 1 h at room temperature. An Immobilon Western Chemiluminescent HRP Substrate Kit (Merck Millipore) was used for detection. Information regarding the primary antibodies can be found in Supplementary Table 4.

### Animal studies

Female nude mice (4-6 weeks old) were purchased from the Shanghai Laboratory Animal Center, Chinese Academy of Sciences. SW620 cells (2 × 10^6^/mice) were subcutaneously injected into mice. When most of the tumors reached approximately 50 mm^3^, the mice were divided into 3 groups based on the tumor volume, and each group of mice were intraperitoneally injected with vehicle (2% DMSO, 5% PEG-400, 5% Tween-80), 2 mg/kg Celastrol or 2 mg/kg 19-048 5 days per week for 2 weeks (totally 10 times). The tumor size was measured using a caliper three times a week, and the tumor volume was calculated with the formula *V* = 1/2 (length × width^2^). After two weeks, the mice were sacrificed and the tumors were collected and photographed. Tumor tissues were embedded in paraffin wax, followed by immunohistochemistry. Animal experiments were handled with all of the relevant ethical regulations related to animal research. The study was approved by the Institutional Animal Care and Use Committee at Shanghai Jiao Tong University School of Medicine.

### Immunohistochemistry

Xenograft tumors from nude mice were fixed in 4% paraformaldehyde for 24 h and embedded in paraffin. Sections were made using paraffin microtomy and baked at 65 °C overnight. After rehydration in a series of graded alcohols, the sections were treated with citrate buffer (pH = 6.0) and hydrogen peroxide. After blocked in 5% BSA for 1 h, the sections were incubated with corresponding primary antibodies (Supplementary Table 4) at 4 °C overnight, followed by incubated with a specific HRP-conjugated secondary antibody and stained with DAB.

### In vivo toxicity test

For acute toxicity test, Nude mice were randomly divided into five groups (four female mice and four male mice in each group). Each group of mice were intraperitoneally injected with vehicle, 10 mg/kg Celastrol, 20 mg/kg Celastrol, 10 mg/kg 19-048 or 20 mg/kg 19-048. The percent survival within 2 weeks was recorded in Supplementary Table 2 and survival curve.

For chronic toxicity test, mice were randomized into three groups. Each group of mice were intraperitoneally injected with vehicle, 2 mg/kg Celastrol or 2 mg/kg 19-048 5 days per week for 2 weeks (totally ten times). Blood samples were collected using EDTA tubes for the routine blood test on day 6 or day 12 after Celastrol or 19-048 treatment. Routine blood test was performed using BC-2800 Vet Automatic animal blood cell analyzer (Mindray).

## Supplementary information

Revised Supplementary - Clean

Revised Supplementary - Marked Up

## Data Availability

The data that support the findings of this study are available from the authors upon reasonable request.
