# Supplementary material for: Celastrol suppresses colorectal cancer via covalent targeting peroxiredoxin 1
Source: Signal Transduct Target Ther. 2023 Feb 3;8:51. doi: 10.1038/s41392-022-01231-4 (PMC9895061; doi:10.1038/s41392-022-01231-4)
Supplement: Supplementary file 1 — Revised Supplementary - Clean [file 41392_2022_1231_MOESM1_ESM.docx]

Supplementary Materials for

**Celastrol suppresses colorectal cancer via covalent targeting peroxiredoxin 1**

**Author information**

Heng Xu^#^, Hongfang Zhao^#^, Chunyong Ding^#^, Defang Jiang, Zijie Zhao, Yang Li, Xiaoyu Ding, Jing Gao, Hu Zhou, Cheng Luo, Guoqiang Chen, Ao Zhang*, Ying Xu*, Hao Zhang*

^#^ These authors contributed equally: Heng Xu, Hongfang Zhao, Chunyong Ding

* Correspondence to: [ao6919zhang@sjtu.edu.cn](mailto:ao6919zhang@sjtu.edu.cn), [yingxuxu@shsmu.edu.cn](mailto:yingxuxu@shsmu.edu.cn), [hao_cadd@simm.ac.cn](mailto:hao_cadd@simm.ac.cn)

**This file includes:**

Chemical Synthesis

Supplementary Figures 1-9

Supplementary Table 1-4

**Chemical Synthesis** Scheme 1. Reagents and conditions: (a) DPPA, DIPEA, toluene, reflux; (b) Et_3_N, THF, rt.

**Methyl (((2*R*,4a*S*,6a*S*,12b*R*,14a*S*,14b*R*)-10-hydroxy-2,4a,6a,9,12b,14a-hexamethyl-11-oxo-1,2,3,4,4a,5,6,6a,11,12b,13,14,14a,14b-tetradecahydropicen-2-yl)carbamoyl)-*L*-phenylalaninate (Compound 19-266).**

The solution of compound **19-047** (40 mg, 0.09 mmol), *L*-phenylalaninemethyl ester hydrochloride (39 mg, 0.18 mmol), Et_3_N (25 μL, 0.18 mmol) in THF (1 mL) was stirred at room temperature overnight. After the completion of the reaction, the mixture was concentrated under vacuum to give a dark red residue, which was purified by column chromatography (DCM/MeOH = 20:1) to afford **19-266** as a red solid (20 mg, 41%). Melting point: 151.9-155.7 °C; ^1^H NMR (400 MHz, CDCl_3_) δ 7.19 (dd, *J* = 13.8, 7.2 Hz, 3H), 7.08 – 6.94 (m, 4H), 6.50 (s, 1H), 6.34 (d, *J* = 7.1 Hz, 1H), 4.65 (q, *J* = 6.4 Hz, 1H), 4.61 – 4.52 (m, 1H), 4.13 – 4.04 (m, 1H), 3.59 (s, 3H), 3.11 – 2.93 (m, 2H), 2.81 (d, *J* = 13.7 Hz, 1H), 2.21 (s, 3H), 2.09 (d, *J* = 13.2 Hz, 1H), 1.96 – 1.85 (m, 2H), 1.84 – 1.75 (m, 2H), 1.73 (d, *J* = 3.9 Hz, 1H), 1.70 – 1.62 (m, 4H), 1.54 – 1.44 (m, 3H), 1.42 (s, 3H), 1.37 (s, 3H), 1.25 (s, 3H), 1.09 (s, 3H), 0.93 (d, *J* = 13.9 Hz, 1H), 0.70 (s, 3H).^13^C NMR (126 MHz, CDCl_3_) δ 178.43, 173.37, 171.00, 165.11, 156.37, 146.12, 136.42, 134.34, 129.45, 128.53, 127.49, 126.99, 119.54, 118.04, 117.28, 53.73, 52.30, 50.54, 45.26, 44.22, 43.28, 39.36, 38.58, 38.31, 36.53, 36.10, 34.23, 33.54, 31.91, 31.45, 30.55, 29.72, 29.30, 29.00, 22.13, 20.48, 10.40. ESI-MS (m/z) 649.4 [M+Na]^+^; HRMS-ESI（m/z）：calcd for: C_39_H_51_N_2_O_5_, [M+H]^+^: 627.3792, found: 627.3801. HPLC purity: 99.47%.

**Methyl (((2*R*,4a*S*,6a*S*,12b*R*,14a*S*,14b*R*)-10-hydroxy-2,4a,6a,9,12b,14a-hexamethyl-11-oxo-1,2,3,4,4a,5,6,6a,11,12b,13,14,14a,14b-tetradecahydropicen-2-yl)carbamoyl)-*L*-alaninate (Compound 19-048).**

The solution of compound **19-047** (100 mg, 0.22 mmol), L-alanine methyl ester hydrochloride (62 mg, 0.44 mmol), and Et_3_N (62 μL, 0.44 mmol) in THF (2 mL) was stirred at room temperature overnight. After the completion of the reaction, the mixture was concentrated under vacuum to give a dark red residue, which was purified by column chromatography (DCM/MeOH = 30:1) to afford **19-048** as a red solid. (74 mg, 60%). Melting point: 188.1-191.3 °C; 1H NMR (400 MHz, Chloroform-d) δ 7.02 (d, *J* = 7.1 Hz, 1H), 6.97 (s, 1H), 6.51 (s, 1H), 6.35 (d, *J* = 7.2 Hz, 1H), 5.27 (s, 1H), 4.50 – 4.40 (m, 2H), 3.84 (d, *J* = 3.2 Hz, 2H), 3.65 (s, 3H), 3.07 (s, 1H), 2.81 (d, *J* = 13.0 Hz, 1H), 2.21 (s, 3H), 2.10 (d, *J* = 12.8 Hz, 1H), 2.01 – 1.86 (m, 2H), 1.81 (d, J = 14.8 Hz, 2H), 1.76 – 1.70 (m, 1H), 1.70 – 1.60 (m, 4H), 1.56 – 1.46 (m, 3H), 1.41 (s, 3H), 1.38 (s, 3H), 1.26 (s, 3H), 1.11 (s, 3H), 0.96 (d, *J* = 13.0 Hz, 1H), 0.74 (s, 3H).^13^C NMR (126 MHz, CDCl_3_) δ 178.45, 174.93, 171.01, 165.14, 156.55, 146.12, 134.35, 127.51, 119.51, 118.05, 117.29, 52.45, 50.47, 48.56, 45.28, 44.21, 43.28, 39.36, 38.33, 36.53, 36.25, 34.23, 33.50, 31.91, 31.45, 30.58, 29.72, 29.25, 29.01, 22.14, 20.52, 19.07, 10.41. ESI-MS (m/z) 573.6 [M+Na]^+^; HRMS-ESI (m/z): calcd for: C_33_H_47_N_2_O_5_, [M+H]^+^: 551.3479, found: 551.3485. HPLC purity: 98.07%.

NMR data for compound **19-266**

^1^H-NMR spectrum for 19-266 recorded in CDCl_3_

^13^C-NMR spectrum for 19-266 recorded in CDCl_3_

HPLC spectrum for compound **19-266**


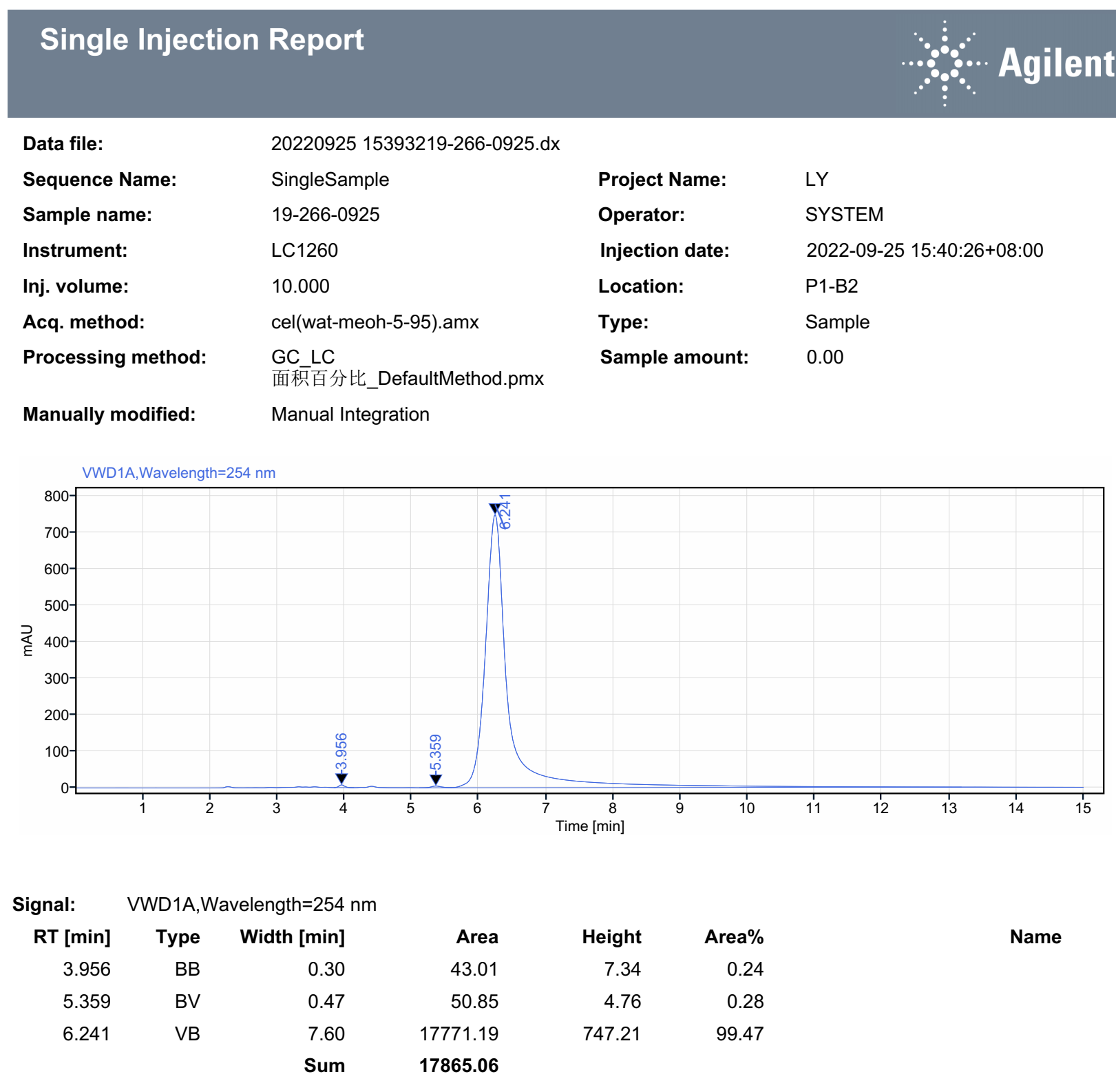


NMR data for compound **19-048**

^1^H-NMR spectrum for 19-048 recorded in CDCl_3_

^13^C-NMR spectrum for 19-048 recorded in CDCl**_3_**

HPLC spectrum for compound **19-048**


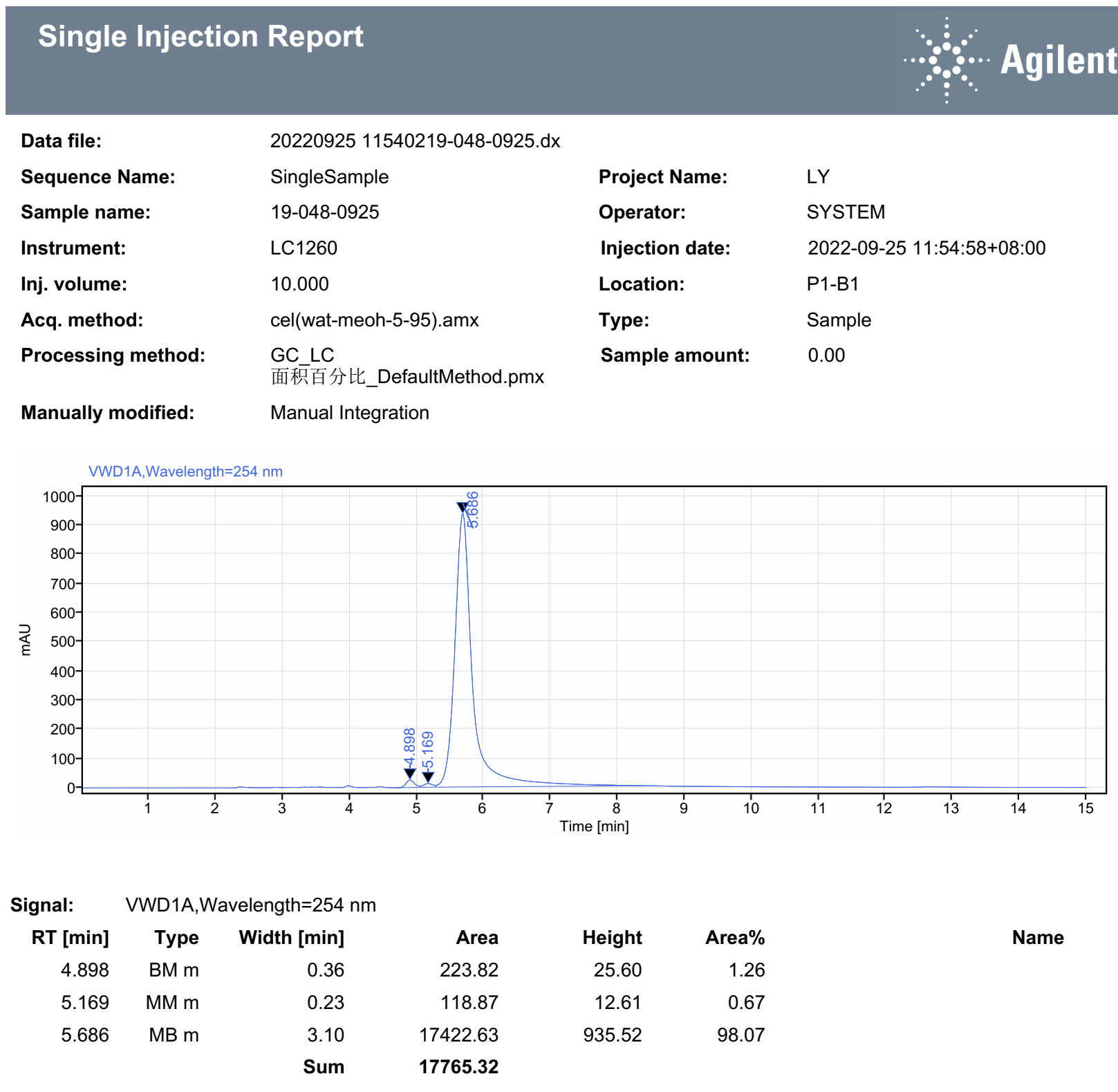


Scheme 2. Synthetic route of alkynylated Celastrol (compound 1).

**(2*R*,4a*S*,6a*S*,12b*R*,14a*S*,14b*R*)-10-hydroxy-2,4a,6a,9,12b,14a-hexamethyl-11-oxo-N-(2-(2-(prop-2-yn-1-yloxy)ethoxy)ethyl)-1,2,3,4,4a,5,6,6a,11,12b,13,14,14a,14b-tetradecahydropicene-2-carboxamide (1).**

**Chemical structure of synthesized alkynylated Celastrol**

To a solution of celastrol (100 mg, 0.22 mmol) in DMF (2 mL) was added HATU (169 mg, 0.44 mmol), DIPEA (193 μL, 1.11mmol), H_2_N-PEG2-Propyne (41 mg, 0.29 mmol) at room temperature. The reaction mixture was stirred at rt overnight and then poured into water and extracted with EA. The combined organic layer was washed with brine, dried over Na_2_SO_4_, and concentrated in vacuo. The residue was purified by silica gel column chromatography to afford compound **1** (53 mg, 42%). Melting point: 64.7-68.0 °C; ^1^H NMR (400 MHz, CDCl_3_) δ 7.00 (dd, *J* = 7.1, 1.4 Hz, 2H), 6.51 (d, *J* = 1.5 Hz, 1H), 6.33 (d, *J* = 7.2 Hz, 1H), 6.27 (t, *J* = 5.4 Hz, 1H), 4.18 (d, *J* = 2.3 Hz, 2H), 3.69 – 3.63 (m, 2H), 3.61 – 3.55 (m, 2H), 3.49 – 3.44 (m, 2H), 3.35 – 3.28 (m, 2H), 2.48 – 2.40 (m, 2H), 2.19 (s, 3H), 2.16 – 2.09 (m, 1H), 2.06 (dd, *J* = 14.4, 3.7 Hz, 1H), 2.00 – 1.93 (m, 1H), 1.91 – 1.78 (m, 4H), 1.72 – 1.58 (m, 4H), 1.56 (d, *J* = 3.1 Hz, 1H), 1.53 – 1.45 (m, 1H), 1.42 (s, 3H), 1.24 (s, 3H), 1.14 (s, 3H), 1.10 (s, 3H), 1.00 (dd, *J* = 14.4, 3.8 Hz, 1H), 0.62 (s, 3H). ^13^C NMR (125 MHz, CDCl_3_) δ 178.47, 177.96, 170.49, 164.92, 146.13, 134.20, 127.50, 119.65, 118.13, 117.21, 79.58, 77.41, 77.16, 76.91, 74.90, 69.97, 69.58, 69.08, 58.50, 45.18, 44.52, 43.14, 40.41, 39.48, 39.16, 38.30, 36.51, 35.05, 33.87, 33.65, 31.75, 31.24, 30.93, 30.17, 29.51, 28.80, 21.86, 18.42, 10.38. HRMS(ESI): calcd for C_36_H_50_NO_5_, [M+H]^+^: 576.3684; found 576.3661. HPLC purity: 97.53%.

**^1^H-NMR**

**^13^C-NMR**

HPLC spectrum for compound **1** (**19-262)**


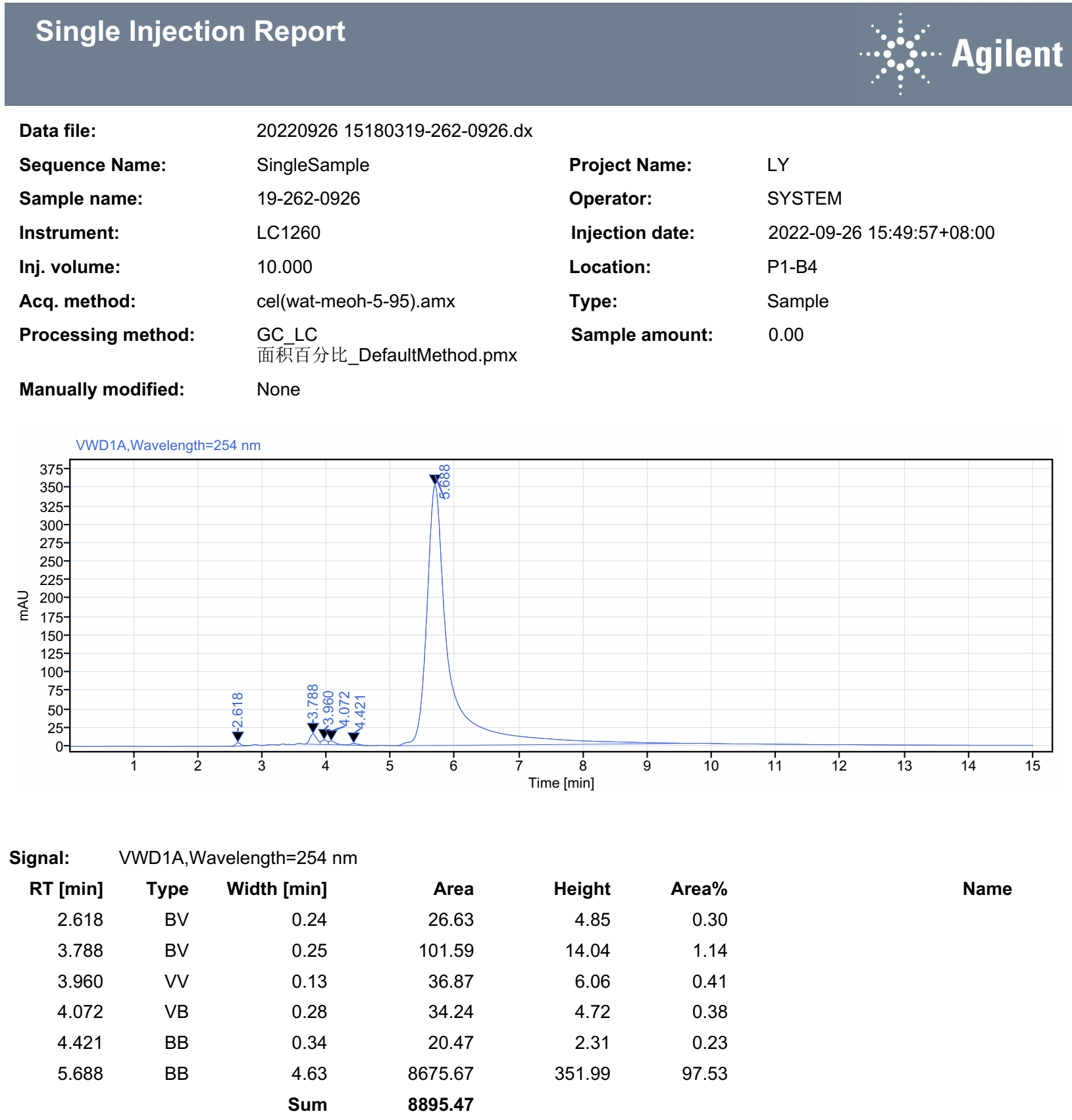


Scheme 3. Synthetic route of alkynylated 19-048 (compound 2).

**But-3-yn-1-yl (((2R,4aS,6aS,12bR,14aS,14bR)-10-hydroxy-2,4a,6a,9,12b,14a-hexamethyl-11-oxo-1,2,3,4,4a,5,6,6a,11,12b,13,14,14a,14b-tetradecahydropicen-2-yl)carbamoyl)-D-alaninate (2)**

**Chemical structure of synthesized alkynylated 19-048**

To a solution of *N*-(*tert*-butoxycarbonyl)-L-alanine (200 mg, 1.06 mmol) in DMF (5 mL) were added EDCI (305 mg, 1.59 mmol), HOBT (215 mg, 1.59 mmol), (2-Hydroxyethyl) acetylene (89 mg, 1.27 mmol). The reaction mixture was stirred at room temperature overnight. The solution was poured into water and extracted with EA three times. The combined organic layer was washed with brine, dried over Na_2_SO_4_, and concentrated in vacuo without further purification to afford crude **a** (160 mg, 63%). To a solution of crude **a** (160 mg, 0.66 mmol) in DCM (5 mL) was added trifluoroacetic acid (2 mL). The reaction mixture was stirred at room temperature for 1 h. The solvent was removed in vacuo. The residue was dissolved in THF (8 mL). And then, Et_3_N (460 μL, 3.31 mmol), compound **c** (228 mg, 0.51 mmol) were added at room temperature. The mixture was stirred at 50 °C for 4 h. The resulting mixture was diluted with EA, washed with brine, dried over Na_2_SO_4_. The organic layer was concentrated in vacuo. The residue was purified by silica gel column chromatography to afford compound **2** (201 mg, 67%). Melting point: 123.4-127.8 °C; ^1^H NMR (400 MHz, CDCl_3_) δ 7.14 – 6.91 (m, 2H), 6.51 (s, 1H), 6.35 (d, *J* = 7.2 Hz, 1H), 4.83 (d, *J* = 8.9 Hz, 1H), 4.45 – 4.35 (m, 1H), 4.30 – 4.09 (m, 3H), 2.87 (d, *J* = 15.0 Hz, 1H), 2.51 (td, *J* = 6.9, 2.6 Hz, 2H), 2.21 (s, 3H), 2.16 – 2.08 (m, 1H), 1.99 (t, *J* = 2.6 Hz, 1H), 1.96 – 1.80 (m, 3H), 1.79 – 1.72 (m, 2H), 1.72 – 1.60 (m, 4H), 1.57 – 1.49 (m, 2H), 1.42 (s, 3H), 1.37 (s, 3H), 1.29 – 1.24 (m, 6H), 1.10 (s, 3H), 0.97 (d, *J* = 13.6 Hz, 1H), 0.74 (s, 3H). ^13^C NMR (125 MHz, CDCl_3_) δ 178.55, 174.40, 170.90, 165.06, 156.39, 146.19, 134.35, 127.51, 119.57, 118.01, 117.30, 79.69, 70.31, 62.87, 50.34, 48.65, 45.29, 44.25, 43.32, 39.41, 38.24, 36.53, 36.31, 34.26, 33.60, 31.89, 31.41, 30.56, 29.77, 29.02, 28.99, 22.15, 20.27, 19.52, 19.00, 10.40. HRMS(ESI): calcd for C_36_H_49_N_2_O_5_, [M+H]^+^: 589.3636; found 589.3652. HPLC purity：96.76%.

**^1^H-NMR**

**^13^C-NMR**

HPLC spectrum for compound **2** (**19-243)**


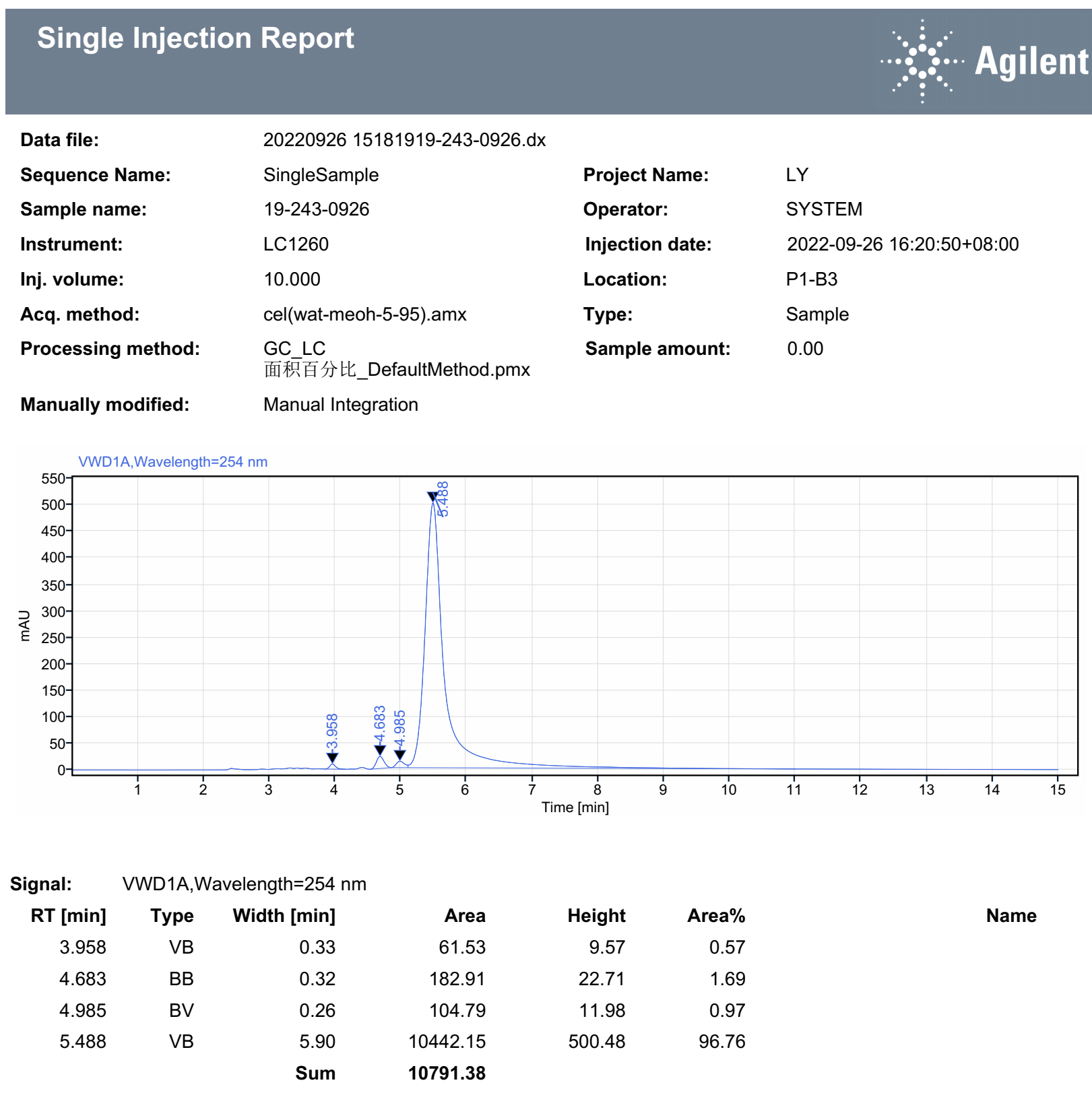


**Supplementary Figures and Tables**

**
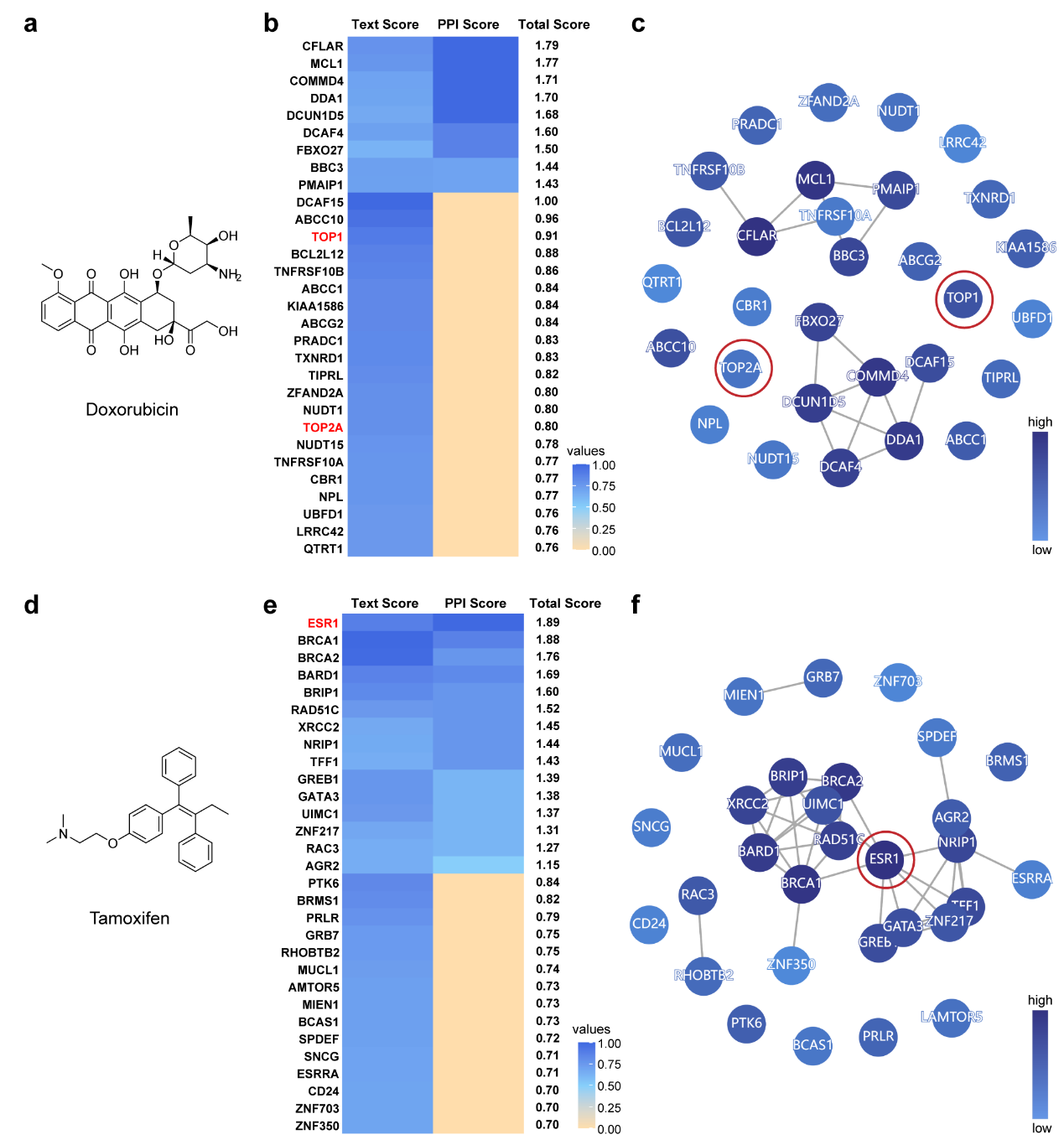
**

**Fig. S1 Test cases for OTTER using approved drugs with known targets.**

**a** Chemical structure of Doxorubicin.

**b** Heatmap for the top-ranking 30 differentially expressed genes using OTTER with the RNA-Seq data from MCF-7 cells treated with Doxorubicin. The known target proteins TOP2A and TOP1 of Doxorubicin ranked 23^rd^ and 12^th^ according to the final scores of text scores plus PPI scores.

**c** The interactive plot generated for the top-ranking 30 differentially expressed genes from Doxorubicin treated cells, after ranking by OTTER with final scores.

**d** Chemical structure of Tamoxifen.

**e** Heatmap for the top-ranking 30 differentially expressed genes using OTTER with the RNA-Seq data from MCF-7 cells treated with Tamoxifen. The known target ESR1 of Tamoxifen ranked 1^st^ according to the final scores of text scores plus PPI scores.

**f** The interactive plot generated for the top-ranking 30 differentially expressed genes from Tamoxifen-treated cells, after ranking by OTTER with final scores.

**
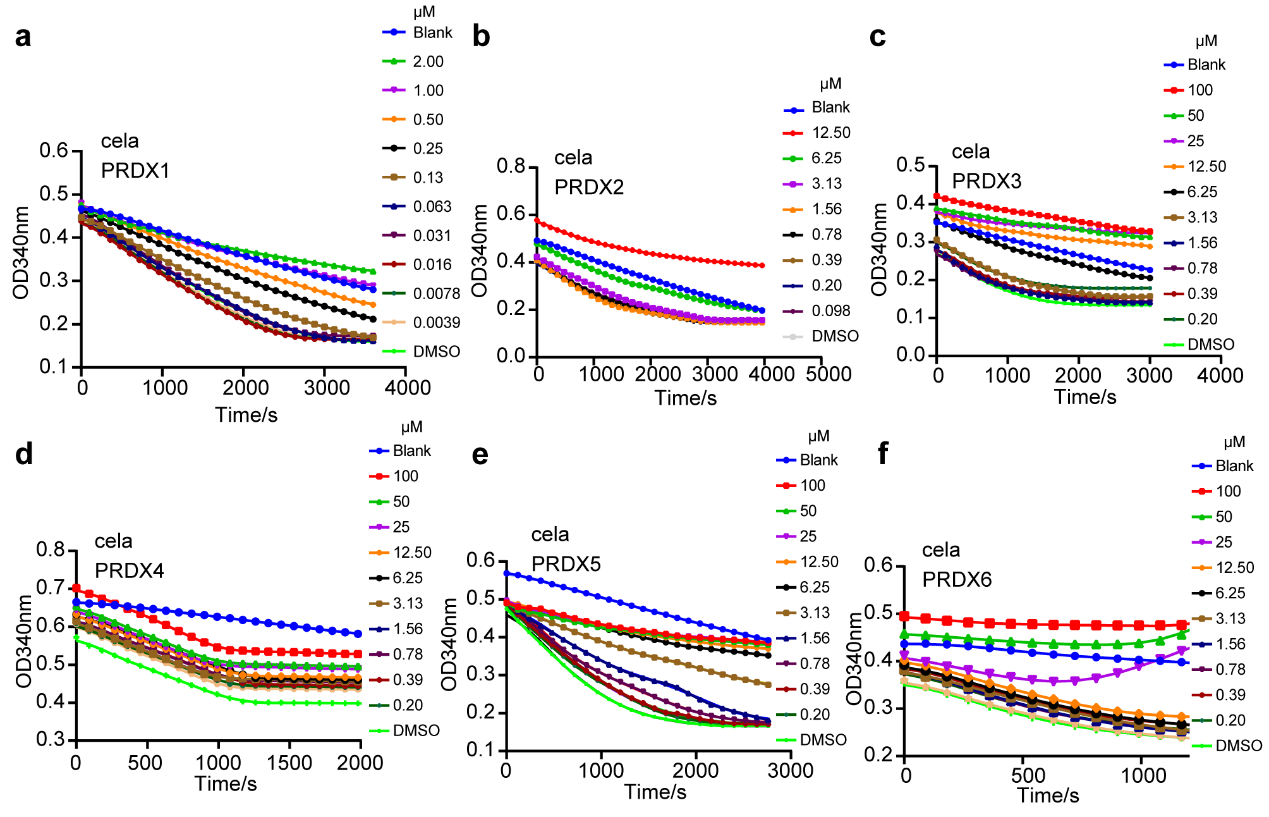
**

**Fig. S2 The peroxidase activity of recombinant PRDX1~PRDX6 with Celastrol incubation was detected using Trx-TrxR-NADPH coupling assay.**

**a-f** Celastrol was incubated with recombinant PRDX1 (**a**), PRDX2 (**b**), PRDX3 (**c**), PRDX4 (**d**), PRDX5 (**e**), or PRDX6 (**f**) for 1.5 h at different concentrations.

The peroxidase activity was monitored with absorbance reduction at 340 nm. For PRDX1, slope of initial 0-2500 s was used to reflect initial reaction rate of each sample. For PRDX2, PRDX3, PRDX5, slope of initial 0-1000 s was used to reflect initial reaction rate. For PRDX4, slope of initial 0-720 s was used to reflect initial reaction rate. For PRDX6, slope of initial 0-480 s was used to reflect initial reaction rate. “Blank” represents assay wells without PRDXs and Celastrol. “DMSO” represents PRDXs incubated DMSO.

**
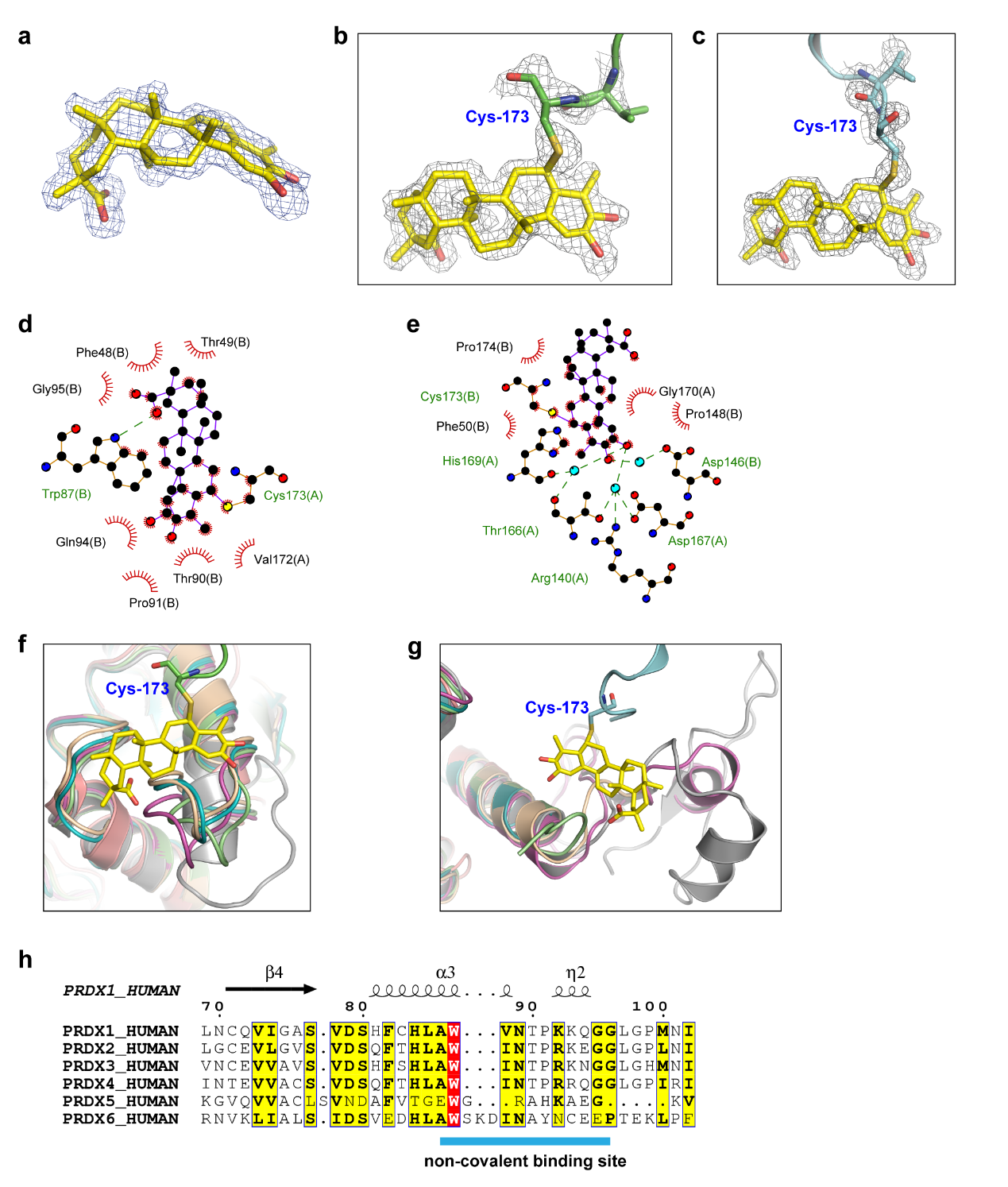
**

**Fig. S3 Crystal structure of PRDX1 in complex with Celastrol.**

**a** The *F*o-*F*c electron density map of Celastrol contoured at 2.5 σ.

**b, c** The 2*F*o-*F*c electron density map contoured at 1.0 σ, for two Celastrol molecules linked to the Cys-173 residues from two adjacent PRDX1 homodimers.

**d, e** Schematic representation of the non-covalent binding sites of two Celastrol molecules with adjacent PRDX1 homodimers analyzed by LigPlot^1^.

**f, g** Conformational difference was observed for the non-covalent binding sites of two Celastrol molecules with adjacent PRDX1 homomers, after structural superposition to PRDX2~PRDX6. PRDX2 (PDB id: 5IJT), PRDX3 (PDB id: 5JCG), PRDX4 (PDB id: 3TJB), PRDX5 (PDB id: 3MNG), and PRDX6 (PDB id: 5B6M).

**h** Sequence alignment of human PRDX protein family by ESPript^2^. The non-covalent binding site of Celastrol containing Gln-94 residue ranges from 86 to 96.


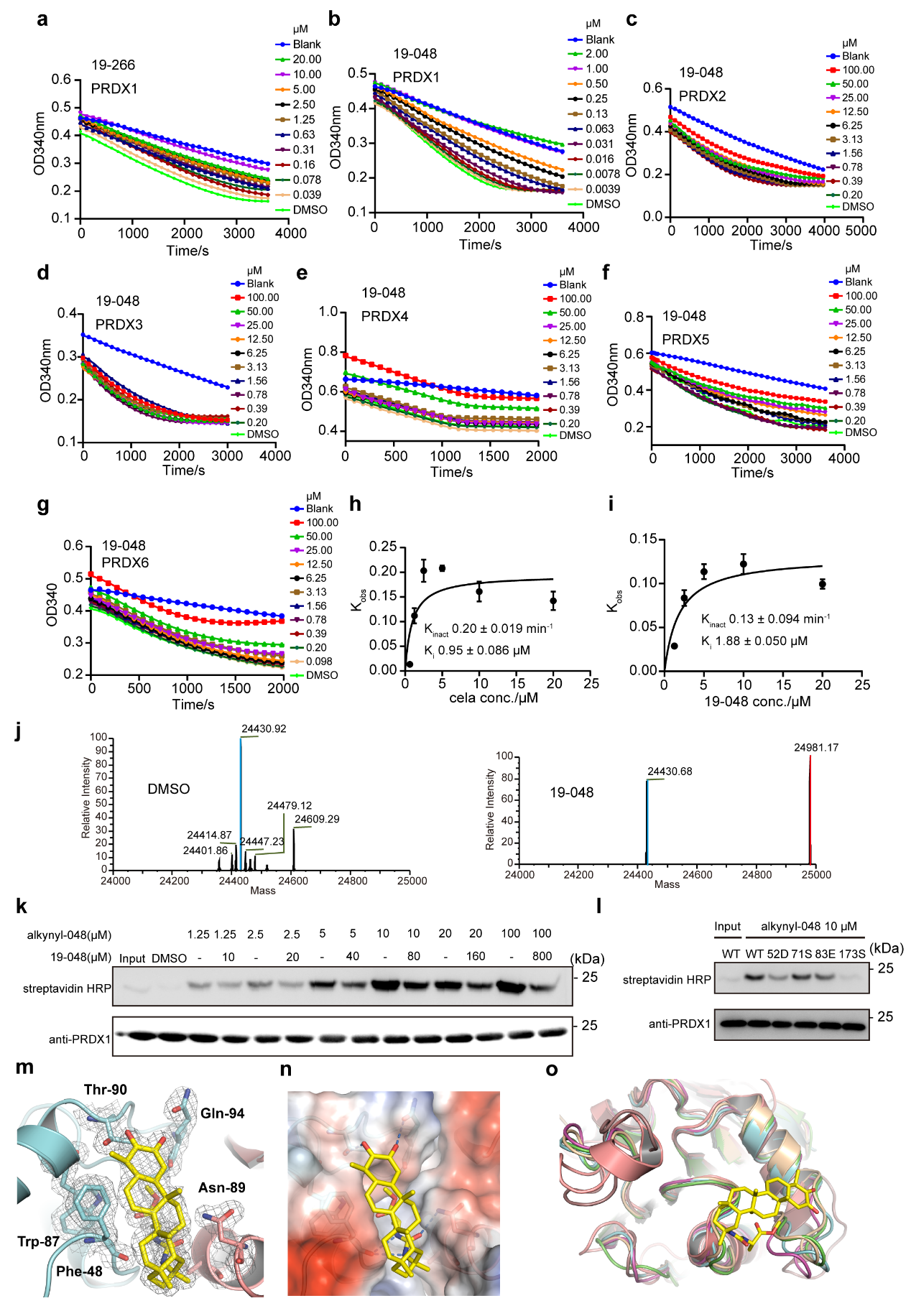


**Fig. S4 New derivative compound of Celastrol named 19-048 showed improved potency and selectivity.**

**a** Compound 19-266 was incubated with recombinant PRDX1 for 1.5 h at different concentrations. The peroxidase activity was monitored with absorbance reduction at 340 nm. Slope of initial 0-2500 s was used to reflect initial reaction rate of each sample. “Blank” represents assay wells without PRDXs and compound 19-266. “DMSO” represents PRDXs incubated with DMSO.

**b-g** Compound 19-048 was incubated with recombinant PRDX1 (**b**), PRDX2 (**c**), PRDX3 (**d**), PRDX4 (**e**), PRDX5 (**f**), or PRDX6 (**g**) for 1.5 h at different concentrations. The peroxidase activity was monitored with absorbance reduction at 340 nm. For PRDX1, slope of initial 0-2500 s was used to reflect initial reaction rate of each sample. For PRDX2, PRDX3, PRDX5, slope of initial 0-1000 s was used to reflect initial reaction rate. For PRDX4, slope of initial 0-720 s was used to reflect initial reaction rate. For PRDX6, slope of initial 0-480 s was used to reflect initial reaction rate. “Blank” represents assay well without PRDXs and compound 19-048. “DMSO” represented PRDXs incubated with DMSO.

**h, i** Determination of K_inact_ and K_i_ of Celastrol (**h**) or compound 19-048 (**i**) for inhibition of PRDX1’s peroxidase activity. Celastrol or compound 19-048 was incubated with recombinant PRDX1 for 0, 20, 40, 60, 80, or 100 min at different concentrations, then the inhibition effect was detected using the Trx-TrxR-NADPH based peroxidase activity assay. Inhibition rates at each incubation time and each compound concentration were calculated from slope of initial reaction.

**j** Mass spectra analysis of recombinant PRDX1 with compound 19-048 binding. The left plot represented PRDX1 incubating with DMSO. The right plot represented PRDX1 with compound 19-048 incubation for 1.5 h. Main mass peak of ligand free PRDX1 was shown in red, while peak of PRDX1-048 complex was shown in cyan. The exact mass of 19-048 is 550.341.

**k** Click chemistry labeling of recombinant PRDX1 through alkynylated 19-048. The experiment and analysis procedures were conducted as in Fig. 2h.

**l** Click chemistry labeling of recombinant wild type PRDX1 and its mutant through alkynylated 19-048. The experiment and analysis procedures were conducted as in Figure 2J.

**m** The 2*F_O_-F_C_* electron density map of compound 19-048 and interacting residues contoured at 1.0 σ. Compound 19-048 and interacting residues were shown as sticks.

**n** Electrostatic potential of binding pocket of compound 19-048. Electropositive area was shown in red surface, while electronegative area was shown in blue. Compound 19-048 was shown as sticks.

**o** Superposition for the crystal structure of compound 19-048 in complex with PRDX1^C52SC83S,1-175aa^ with PRDX2 (PDB id: 5IJT), PRDX3 (PDB id: 5JCG), PRDX4 (PDB id: 3TJB), PRDX5 (PDB id: 3MNG), and PRDX6 (PDB id: 5B6M).

All data is shown in mean ± SEM.


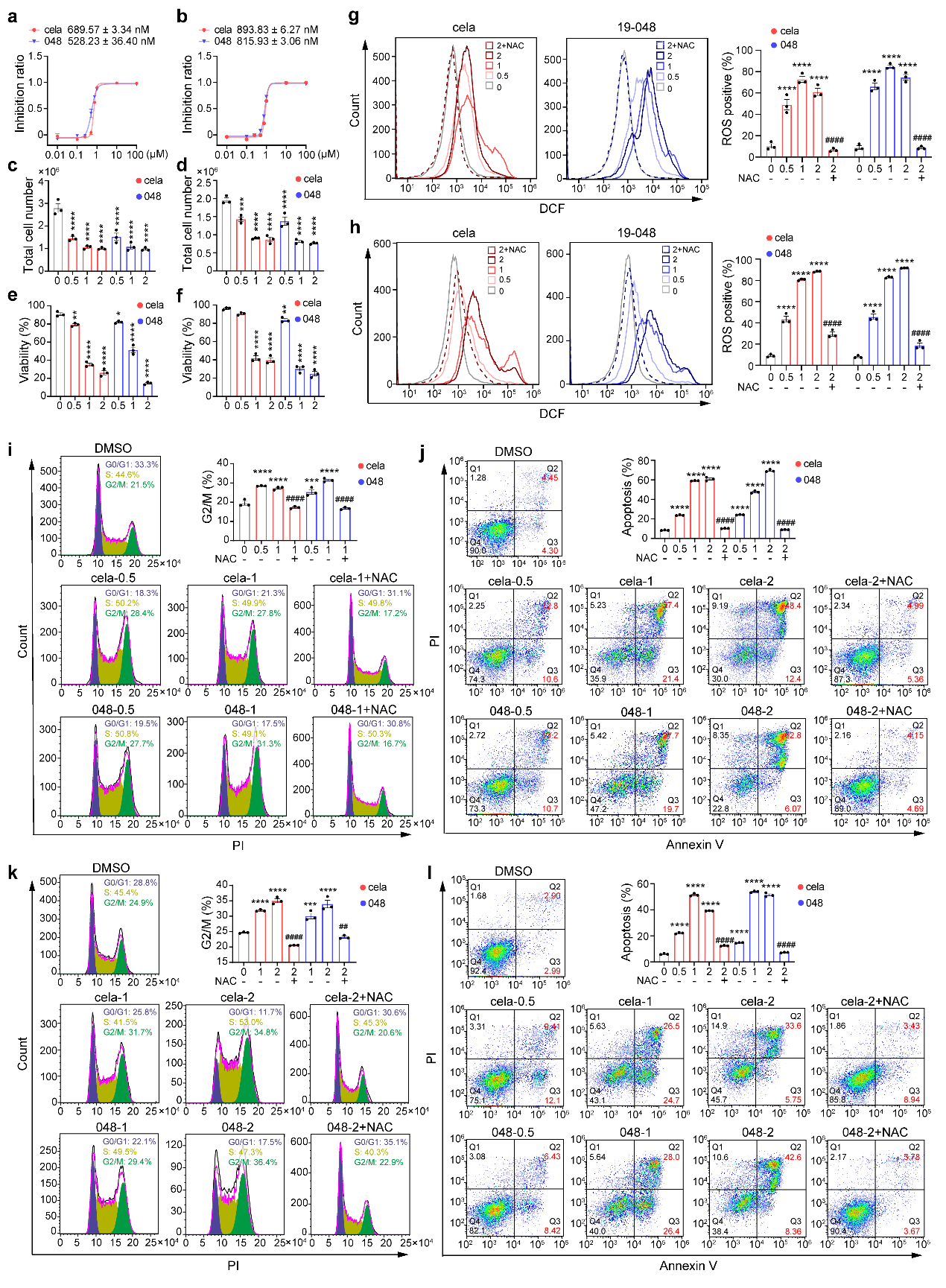


**Fig. S5 Celastrol and compound 19-048 induced cell cycle arrest and apoptosis by increasing ROS in colorectal cancer cells.**

**a, b** Half growth inhibitory concentration (IC_50_) of Celastrol and compound 19-048 were measured in SW620 (**a**) and HCT116 (**b**) cells after treatment with compounds for 24 h.

**c, d** Cell number was measured by trypan-blue staining. SW620 (C) and HCT116 (D) cells were treated with the indicated concentrations (μM) of Celastrol or compound 19-048 for 24 h. ***p < 0.001, ****p < 0.0001 versus vehicle by one-way ANOVA.

**e, f** Cell viability were measured by trypan-blue staining. SW620 (**e**) and HCT116 (**f**) cells were treated with the indicated concentrations (μM) of Celastrol or compound 19-048 for 24 h. *p < 0.05, **p < 0.01, ****p < 0.0001 versus vehicle by one-way ANOVA.

**g, h** ROS measurement of SW620 (**g**) and HCT116 (**h**) cells with compounds treatment. Cells were treated with the indicated concentrations (μM) of Celastrol or compound 19-048 for 24 h. ROS level was analyzed by flow cytometry with DCFH-DA staining. ROS quantification and statistical analysis are shown in histograms. ^####^p < 0.0001 versus 2 μM Celastrol or compound 19-048 without NAC treatment and ****p < 0.0001 versus vehicle by two-way ANOVA.

**i** Cell cycle analysis of SW620 cells with compounds treatment. Cells were treated with the indicated concentrations (μM) of Celastrol or compound 19-048 for 24 h. Cell cycle distribution was detected by flow cytometry with PI staining. Quantification of G2/M phase cycle arrest and statistical analysis are shown in histograms. ^####^p < 0.0001 versus 1 μM Celastrol or compound 19-048 without NAC treatment by Student’s t test. ***p < 0.001, ****p < 0.0001 versus vehicle by one-way ANOVA.

**j** Apoptotic cell analysis of SW620 cells with compounds treatment. Cells were treated with the indicated concentrations (μM) of Celastrol or compound 19-048 for 24 h. Apoptotic cells were measured by flow cytometry with AnnexinV and PI staining. Quantification of apoptotic cells (Annexin V^+^) and statistical analysis are shown in histograms. ^####^p < 0.0001 versus 2 μM Celastrol or compound 19-048 without NAC treatment by Student’s t test. ****p < 0.0001 versus vehicle by one-way ANOVA.

**k** Cell cycle analysis of HCT116 cells with compounds treatment. Cells were treated with the indicated concentrations (μM) of Celastrol or compound 19-048 for 24 h. Detection of cell cycle distribution and statistical quantification analysis were executed as in (**i**). ^##^p < 0.01, ^####^p < 0.0001 versus 2 μM Celastrol or compound 19-048 without NAC treatment by Student’s t test. ***p < 0.001, ****p < 0.0001 versus vehicle by one-way ANOVA.

**l** Apoptotic cell analysis of HCT116 cells with compounds treatment. Cells were treated with the indicated concentrations (μM) of Celastrol or compound 19-048 for 24 h. Measurement of apoptotic cells and statistical quantification analysis were executed as in (**j**). ^####^p < 0.0001 versus 2 μM Celastrol or compound 19-048 without NAC treatment by Student’s t test. ****p < 0.0001 versus vehicle by one-way ANOVA.

Data were calculated from triplicate experiments and presented as mean ± SEM. Vehicle: control group without NAC and compound treatment.


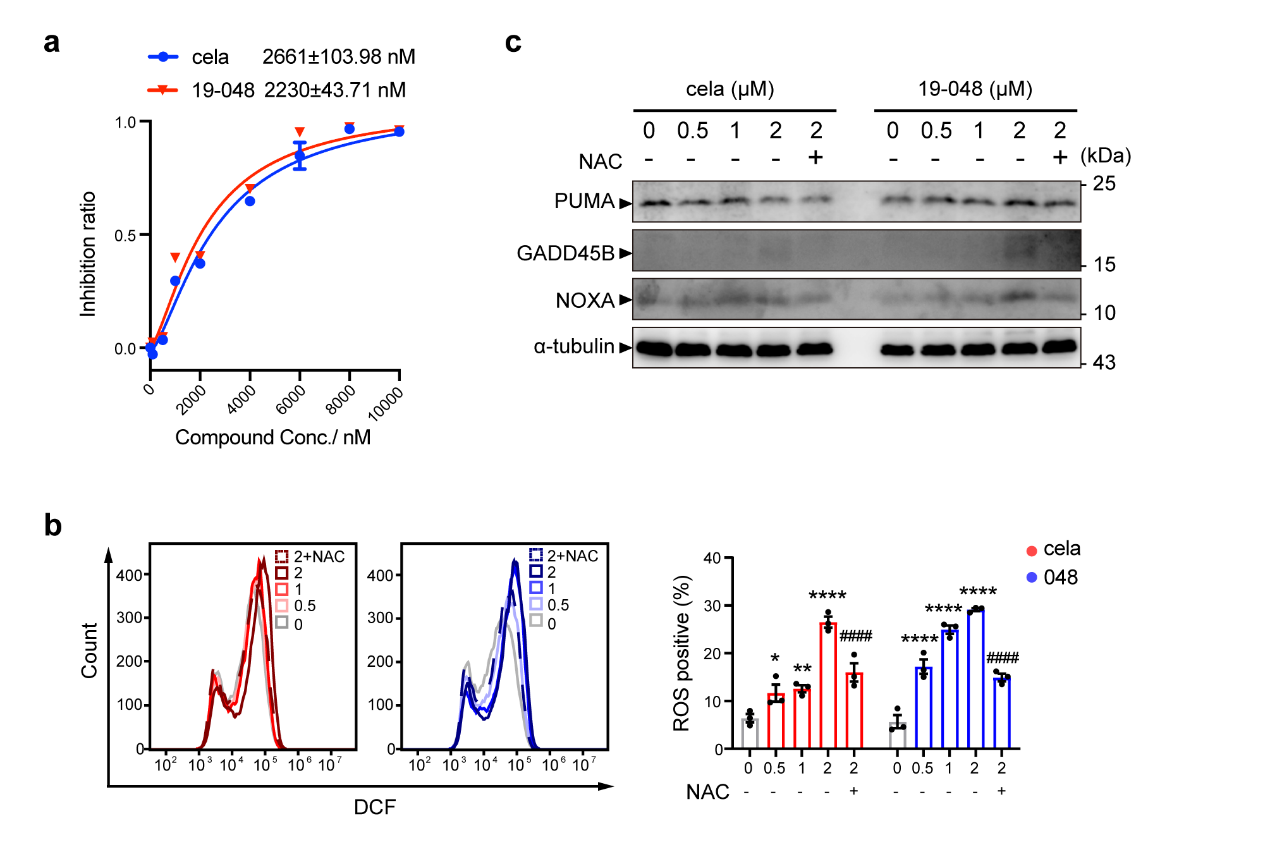


**Fig. S6 Celastrol and compound 19-048 effect on normal human colon mucosal epithelial cell line NCM460.**

**a** Half growth inhibitory concentration (IC_50_) of Celastrol and compound 19-048 were measured in NCM460 cells after treatment with compounds for 24 h.

**b** ROS measurement of NCM460 cells with compounds treatment. Cells were treated with the indicated concentrations (μM) of Celastrol or compound 19-048 for 24 h. ROS level was analyzed by flow cytometry with DCFH-DA staining. ROS quantification and statistical analysis are shown in histograms. ^####^p < 0.0001 versus 2 μM Celastrol or compound 19-048 without NAC treatment and *p < 0.05, **p < 0.01, ****p < 0.0001 versus vehicle by two-way ANOVA.

**c** Protein expression level analysis of p53 target genes in NCM460. Cells were treated with the indicated concentrations (μM) of Celastrol or compound 19-048 for 24 h. Proteins were analyzed by western blot. NAC was used at the concentration of 5 mM.


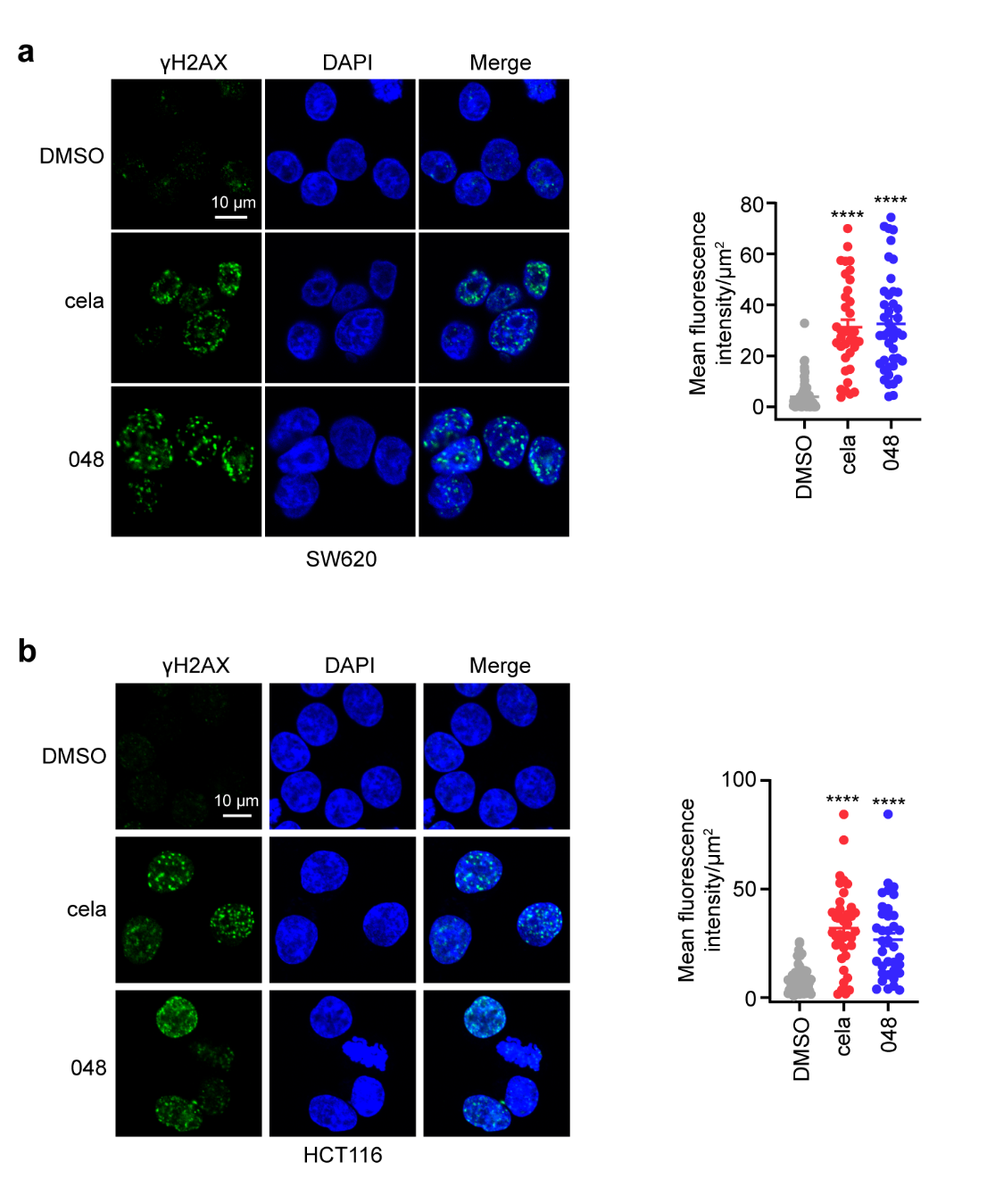


**Fig. S7 Celastrol and compound 19-048 induced DNA damage in colorectal cancer cells.**

**a, b** Immunofluorescence staining of γH2AX and DAPI in SW620 (**a**) and HCT116 (**b**). Cells were treated with 1 μM Celastrol or compound 19-048 for 24 h. Data are presented as mean ± SEM. ****p < 0.0001 versus DMSO control by Student’s t test.


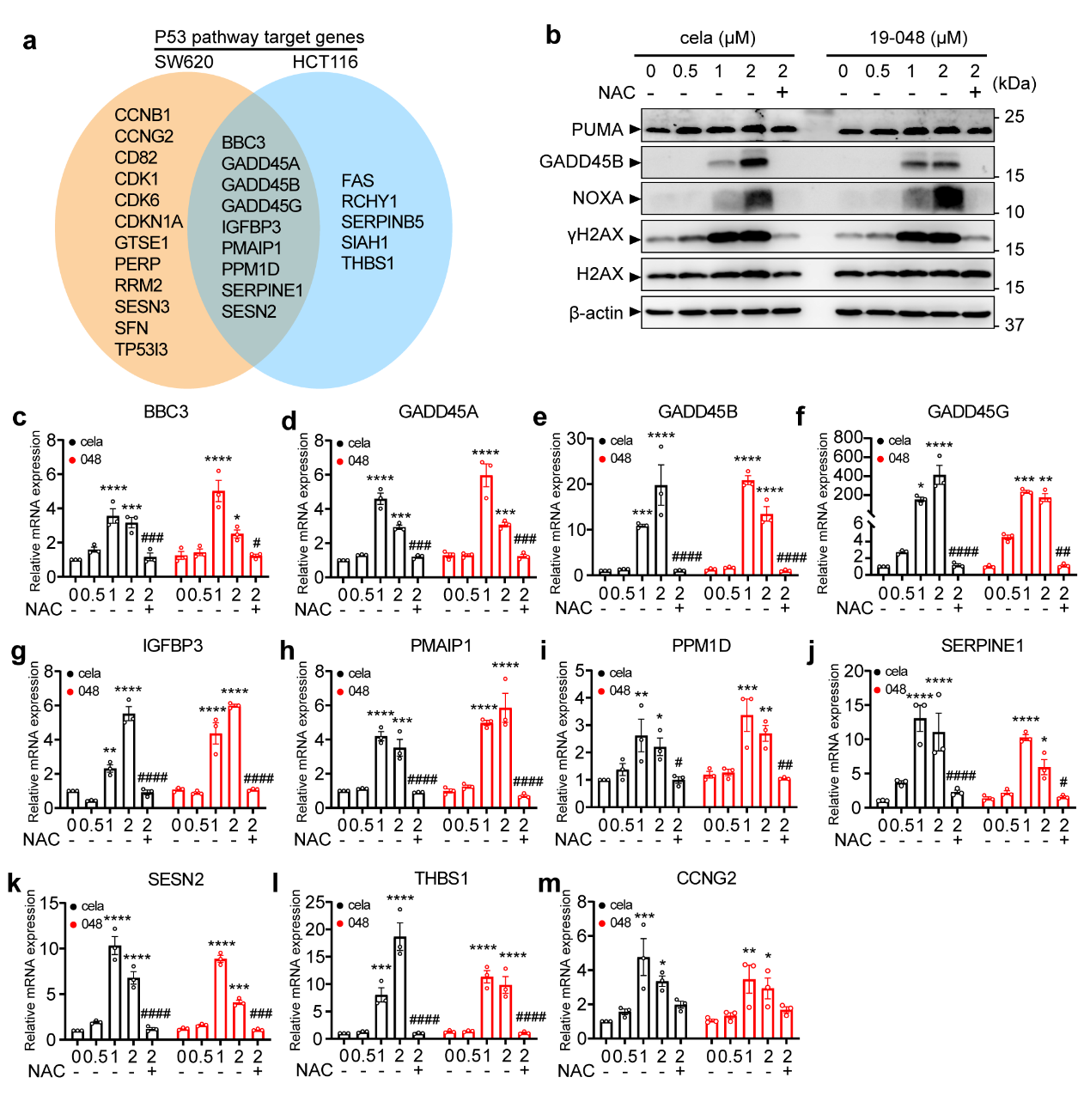


**Fig. S8 Transcriptome-wide RNA sequencing to identify related signaling pathways and genes regulated by Celastrol and compound 19-048 in colorectal cancer cells.**

**a** Venn diagram showing DEG names from the p53 signaling pathway in SW620 and HCT116 cells treated with Celastrol or compound 19-048 for 24 h.

**b** Protein expression level analysis of p53 target genes and DNA damage marker in HCT116 (as in Fig. 6o). Cells were treated with the indicated concentrations (μM) of Celastrol or compound 19-048 for 24 h. Proteins were analyzed by western blot. NAC was used at the concentration of 5 mM.

**c-m** Transcriptional regulation of p53 target genes with celastrol or 19-048 treatment was measured in HCT116. Cells were treated with the indicated concentrations (μM) of Celastrol or compound 19-048 for 24 h. Indicated genes were analyzed by qRT-PCR. NAC was used at the concentration of 5 mM. Data were calculated from triplicate experiments and presented as mean ± SEM. Statistical significance was determined by two-way ANOVA. *p < 0.05, **p < 0.01, ***p < 0.001, ****p < 0.0001 versus vehicle. ^#^p < 0.05, ^##^p < 0.01, ^###^p < 0.001, ^####^p < 0.0001 versus 2 μM Celastrol or compound 19-048 without NAC treatment. Vehicle: control group without NAC and compound treatment.


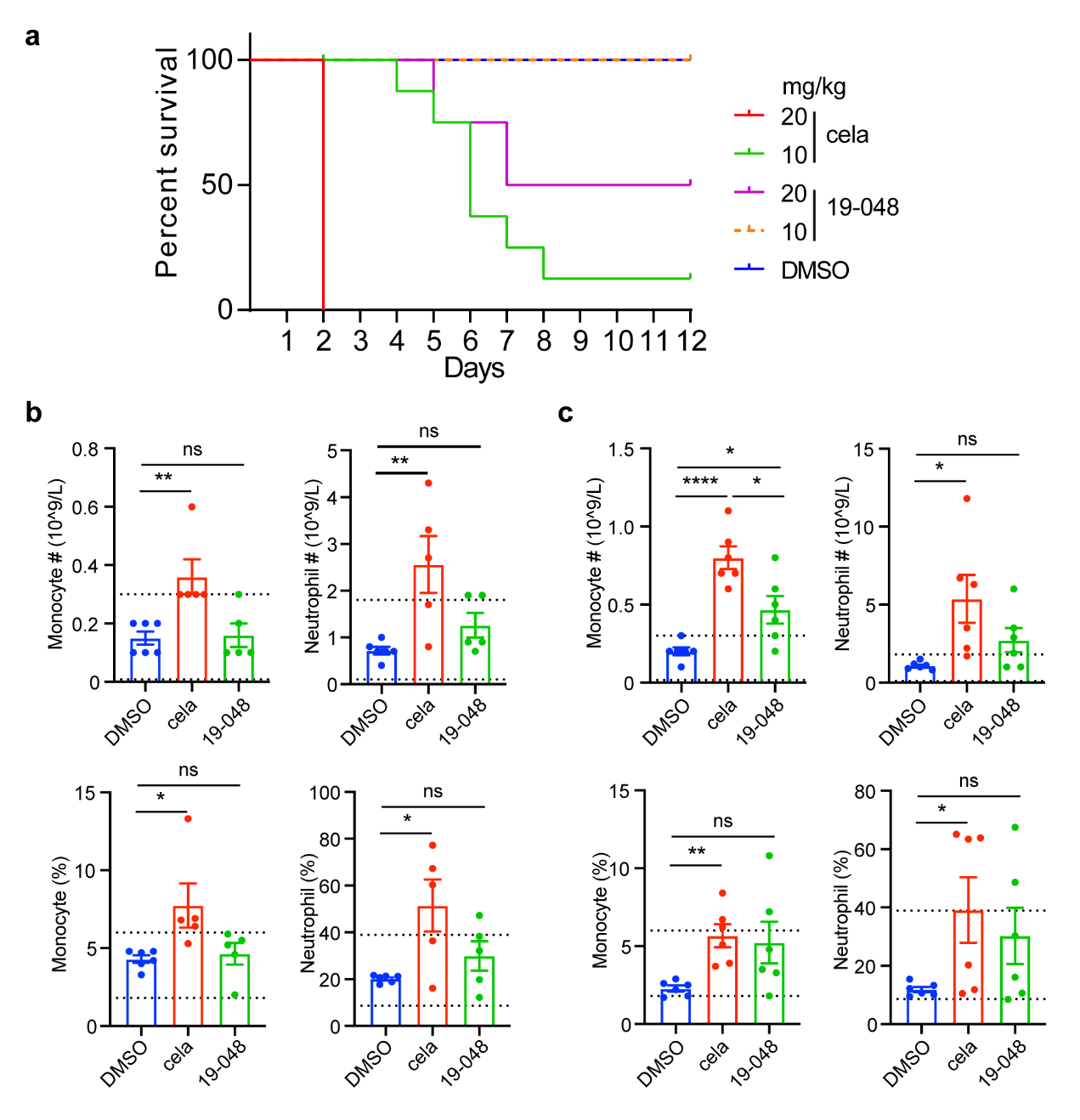


**Fig. S9** **Compound 19-048 reduced *in vivo* toxicity compared to Celastrol.**

**a** Acute toxicity survival curves of Celastrol and 19-048. Nude mice were treated with Celastrol or compound 19-048 on day 1 with the dose of 20 mg/kg or 10 mg/kg. n = 8 per group.

**b, c** Routine blood test of Celastrol and 19-048 in chronic toxicity test. Nude mice were treated with Celastrol or compound 19-048 (2 mg/kg, five times per week for two weeks) or DMSO control. Blood samples were collected for the routine blood test on day 6 (**b**) and day 12 (**c**) after Celastrol or 19-048 treatment. Dotted lines indicate reference intervals. Data are shown as mean ± SEM, statistical significance was determined by student’s t-test.

**Table S1. Data statistics of X-Ray data processing and refinement for Celastrol and compound 19-048 in complex with PRDX1^C52SC83S,1-175aa^.**

|  | **PRDX1-Celastrol**  (PDB: **7WET**) | **PRDX1-19-048**  (PDB: **7WEU**) |
| --- | --- | --- |
| **Date Collection** |  |  |
| Wavelength (Å) | 0.9785 | 0.9785 |
| Space group | P 21 21 21 | P 21 21 21 |
| Cell dimensions |  |  |
| *a, b, c* (Å) | 64.6, 78.4, 81.2 | 63.9, 77.8, 81.2 |
| α, β, γ (°) | 90, 90, 90 | 90, 90, 90 |
| Resolution (Å) | 30.02 - 1.76 (1.82 - 1.76) ^*^ | 35.98 - 1.81 (1.88 - 1.81) ^*^ |
| *R*_merge_ | 0.057 (0.306) | 0.063 (0.206) |
| *I / σI* | 36.34 (7.86) | 33.54 (11.74) |
| Completeness (%) | 99.85 (99.90) | 99.94 (100.00) |
| Multiplicity | 13.0 (12.9) | 13.3 (13.3) |
|  |  |  |
| **Refinement** |  |  |
| Resolution (Å) | 30.02 - 1.76 | 35.98 - 1.81 |
| No. reflections | 41,454 (4,091) | 37,334 (3,669) |
| *R*_work_ */ R*_free_ | 0.156 / 0.180 | 0.156 / 0.195 |
| Number of atoms |  |  |
| Protein | 2746 | 2656 |
| Solvent | 448 | 446 |
| Protein residues | 345 | 334 |
| B-factors (Å^2^) |  |  |
| Protein | 19.06 | 18.98 |
| Solvent | 32.09 | 31.90 |
| Ramachandran |  |  |
| Favored (%) | 98.23 | 98.18 |
| Allowed (%) | 1.77 | 1.82 |
| Outliers (%) | 0.00 | 0.00 |
| R.m.s. deviations |  |  |
| Bond lengths (Å) | 0.007 | 0.006 |
| Bond angles (°) | 1.19 | 0.85 |

* Values in parentheses are for highest-resolution shell. Data was obtained from a single crystal.

**Table S2. Percent survival of acute toxicity experiment.**

| Compound | Dose (mg/kg) | Male (n=4) | | | Female (n=4) | |
| --- | --- | --- | --- | --- | --- | --- |
|  |  | Survival number | Survival rate (%) | Survival number | | Survival rate (%) |
| DMSO | / | 4 | 100 | 4 | | 100 |
| Celastrol | 10 | 1 | 25 | 0 | | 0 |
| Celastrol | 20 | 0 | 0 | 0 | | 0 |
| 19-048 | 10 | 4 | 100 | 4 | | 100 |
| 19-048 | 20 | 4 | 100 | 0 | | 0 |

**Table S3. List of primers used in quantitative real-time PCR.**

| **Gene Name** | **Forward** | **Reverse** |
| --- | --- | --- |
| BBC3 | GACCTCAACGCACAGTACGAG | AGGAGTCCCATGATGAGATTGT |
| GADD45A | GAGAGCAGAAGACCGAAAGGA | CAGTGATCGTGCGCTGACT |
| GADD45B | TACGAGTCGGCCAAGTTGATG | GGATGAGCGTGAAGTGGATTT |
| GADD45G | CAGATCCATTTTACGCTGATCCA | TCCTCGCAAAACAGGCTGAG |
| IGFBP3 | AGAGCACAGATACCCAGAACT | GGTGATTCAGTGTGTCTTCCATT |
| PMAIP1 | ACCAAGCCGGATTTGCGATT | ACTTGCACTTGTTCCTCGTGG |
| PPM1D | CTGTACTCGCTGGGAGTGAG | GTTCGGGCTCCACAACGATT |
| SERPINE1 | ACCGCAACGTGGTTTTCTCA | TTGAATCCCATAGCTGCTTGAAT |
| SESN2 | CCTCTGGGCGAGTAGACAAC | GGAGCCTACCAGGTAAGAACA |
| THBS1 | AGACTCCGCATCGCAAAGG | TCACCACGTTGTTGTCAAGGG |
| CCNG2 | TCTCGGGTTGTTGAACGTCTA | GTAGCCTCAATCAAACTCAGCC |

**Table S4. List of antibodies used in this study.**

| **Antibody** | **Company** | **Dilution** | **Catalogue number** | **Clone number** |
| --- | --- | --- | --- | --- |
| Peroxiredoxin 1 | Abcam | 1:1000 | ab41906 |  |
| GADD45B | Abcam | 1:500 | ab230646 |  |
| GADD45G | Abcam | 1:400 | ab196774 |  |
| NOXA | Abcam | 1:1000 | ab13654 |  |
| PUMA | Abcam | 1:500 | ab9645 |  |
| Phospho-Histone H2A.X (Ser139) | Cell Signaling Technology | 1:1000 | 9718 | 20E3 |
| Histone H2A.X | Cell Signaling Technology | 1:1000 | 7631 | D17A3 |
| Beta Actin | Proteintech | 1:5000 | 60008-1-Ig | 7D2C10 |
| Anti-mouse IgG, HRP-linked Antibody | Cell Signaling Technology | 1:2000 | 7076 |  |
| Anti-rabbit IgG, HRP-linked Antibody | Cell Signaling Technology | 1:2000 | 7074 |  |
| Donkey anti-Rabbit IgG, Alexa Fluor 488 | Thermo Fisher Scientific | 1:1000 | A-21206 |  |
| 4′,6-diamidino-2-phenylindole (DAPI) | Vector Laboratories | 1:1000 | H-1200 |  |

**Reference**

1 Wallace, A. C., Laskowski, R. A. & Thornton, J. M. LIGPLOT: a program to generate schematic diagrams of protein-ligand interactions. *Protein Eng.* **8**, 127-134 (1995).

2 Robert, X. & Gouet, P. Deciphering key features in protein structures with the new ENDscript server. *Nucleic Acids Res.* **42**, W320-W324 (2014).
